# The arrival of millets to the Atlantic coast of northern Iberia

**DOI:** 10.1038/s41598-022-23227-4

**Published:** 2022-11-03

**Authors:** Borja González-Rabanal, Ana B. Marín-Arroyo, Emanuela Cristiani, Andrea Zupancich, Manuel R. González-Morales

**Affiliations:** 1 Grupo de I+D+I EVOADAPTA (Evolución Humana y Adaptaciones durante la Prehistoria), Dpto. Ciencias Históricas, Universidad de Cantabria, Av. Los Castros 44, 39005 Santander, Spain; 2 DANTE-Diet and ANcient TEchnology Laboratory, Department of Oral and Maxillo-Facial Sciences, Sapienza University of Rome, Via Caserta 6, 00161 Rome, Italy; 3 Archaeology of Social Dynamics, Institución Milá y Fontanals, Spanish National Research Council (CSIC), Barcelona, Spain; 4 Instituto Internacional de Investigaciones Prehistóricas de Cantabria (IIIPC), Universidad de Cantabria-Gobierno de Cantabria, Santander, Spain

**Keywords:** Ecology, Evolution, Plant sciences

## Abstract

Despite being one of the most important crops in the recent prehistory of Eurasia, the arrival and exploitation of millets in the westernmost part of Europe are still largely underexplored. Here and for the first time, we report multipronged biomolecular evidence of millet consumption along the Atlantic façade of northern Iberia through a combination of radiocarbon dating, stable isotopes, and dental calculus analyses on the human individuals found in the burial site of El Espinoso cave (Asturias, Spain). The high-resolution chronological framework established for individuals placed the burials between 1235 and 1099 cal. BC. The discovery of high δ^13^C values on their bone collagen and the identification of polyhedral starch grains within their dental plaque underline the relevance of C_4_ plants in their diet and highlights the timing of the systematic consumption of millets in the Late Bronze Age. Our data support previous regional archaeobotanical evidence and establish a more precise chronology of the dispersal of millets into northern Iberia during the Bronze Age, becoming an essential crop until the arrival of maize from America after AD 1492. This study emphasizes the importance of multidisciplinary methods to ascertain the origin and development of agricultural practices during recent prehistory.

## Introduction

Millets are small-seed spring/summer domestic grasses that played a key role in human diet and culture across Europe by the first-millennium cal. BC^1^. Two millet species have been traditionally cultivated in Europe during recent prehistory: *Setaria italica* (foxtail millet) and *Panicum miliaceum* (broomcorn millet)^2^. These plants have a short lifecycle^3^, allowing for annual crop rotation with other cereals, like wheat or barley^4^. Millets are crops with great ecological adaptability to different altitudes, soils, and climates^5^ and a rich nutritional value^6^. They are used to feed both people and animals^7^, and as ingredient in diverse alcohol fermentation techniques^8^. Unfortunately, despite their relevance among farming societies during the Iron Age, little is known about when and how millets were introduced into western Europe, specifically in northern Atlantic Iberia^9^.

The earliest evidence for domesticated broomcorn and foxtail millets comes from north-eastern China and eastern Inner Mongolia, dating to around 6000–5500 cal. BC^10–12^, although broomcorn millet has slightly earlier chronologies than foxtail millet in these areas. From East and Central Asia, the spread of millets would have occurred in the late third millennium cal. BC across the Inner Asian Mountain Corridor^13^ and the Eurasian Steppes^14^. In West Asia and East Europe, the spread of millet cultivation took place during the second millennium cal. BC, producing the first episode of food globalization which laid the foundations of an interconnected Eurasian continent^15^. In these areas, mobile pastoralists might have constituted key agents in the diffusion of these crops^16^ based on the coexistence of livestock and plant cultivation practices across Europe^17^. Direct radiocarbon dates obtained from several broomcorn millet grains, recovered at different Central and Eastern European sites, confirm that millet cultivation arrived in these regions during the second half of the second millennium cal. BC^18–20^, a considerably younger chronology than what was previously thought^21^. Instead, for western regions such as Britain, the latest direct radiocarbon dates point to a slightly later arrival around the first half of the first millennium cal. BC^22^.

Millets have been traditionally seen as minor crops, with a secondary role in past human economies, used as food for people of low social status and/or as fodder for animals^23^. Their small-seed morphology made them hard to find in old excavations, but the implementation of intensive collection programs and the systematic use of flotation systems during recent decades^24^ have favored the recovery of millet seeds, significantly enriching the archaeobotanical record worldwide. Ethnographic and experimental studies have also improved knowledge about millet cultivation, including harvesting, threshing, sieving, milling, storage, and cooking activities^25,26^. Furthermore, comparisons of nutritional components of millets show a similar, and even higher, dietary intake in terms of proteins, minerals, and vitamins than other cereals crops^27^. Recently, ancient starches and phytoliths retrieved in dental calculus and ground stone tool surfaces have provided new direct and indirect evidence for the prehistoric consumption of millets, given their potential to be differentiated at the *genus* or species level^28–30^ and between wild or domestic species^31,32^. Simultaneously, isotopic evidence for C_4_ plant consumption has been reported in different European regions, questioning the relevance of millets in their diets during the Bronze Age^33^. Thus, the current evidence about millet consumption is continuously growing, and millets might have been more relevant than previously thought in the recent prehistory.

In northern Iberia, the Cantabrian region has yielded an extraordinary Bronze Age funerary record where caves were selected as burial locations by early farmers^34^. Despite the richness of these assemblages, biomolecular approaches have rarely been applied to explore evidence related to the origin and development of its regional agriculture, such as the timing of the arrival of millets and how the human populations exploited them. Until the 1990s, millet cultivation was considered to be an Iron Age innovation associated with the hillfort culture^35^. The currently available carpological data support the presence of broomcorn and foxtail millets in this area, at least, since the Late Bronze Age, with some limited evidence from the Middle Bronze Age^9^. However, no direct dates on those seeds confirm their exact chronology. Similarly, stable isotope analysis of bone collagen from Bronze Age individuals failed to detect isotopic signals related to C_4_ plant consumption. Moreover, dental calculus analysis has never been applied to this spatio-temporal range that would allow identifying millet species' starch grains.

With the aim to identify the millet consumption in the Atlantic coast of northern Iberia and to ascertain when and how these crops were systematically exploited during the Bronze Age of this region, this paper combines direct radiocarbon dating on human remains, stable isotope analysis (δ^13^C, δ^15^N and δ^34^S) on human and animal bone collagen, and the study of starch grains entrapped in the dental calculus of humans found at El Espinoso burial cave.

### El Espinoso cave

**Figure 1:**
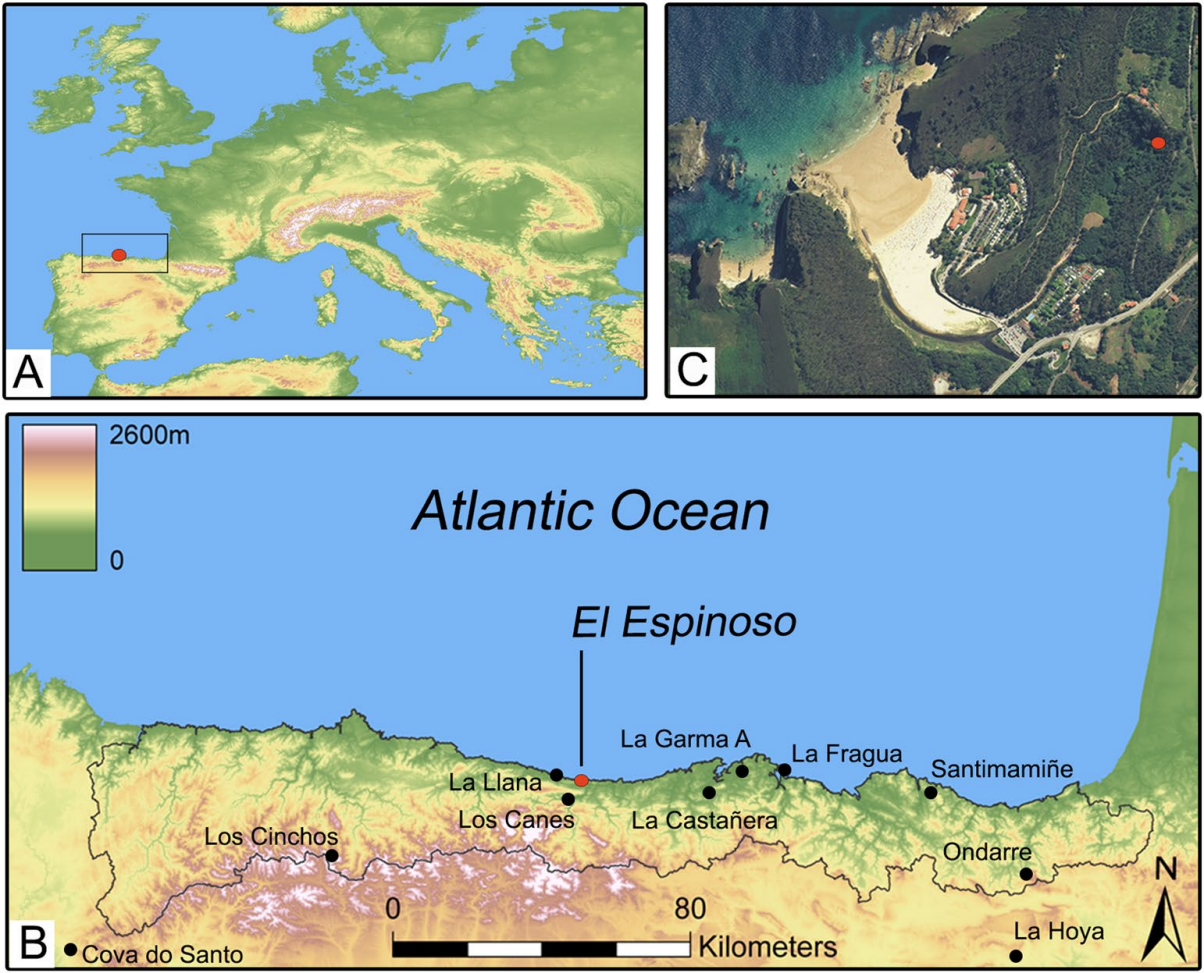
Location of El Espinoso cave in the Atlantic façade of southwestern Europe (**A**) and the Cantabrian Region (northern Iberia) marked in black (**B**). (**C**) Aerial photography of the site location. Maps of (**A**) and (**B**) were generated using ArcGis Pro software (v2.4.0 https://arcgis.com/). The original base maps were extracted from the SRTM Data web site (https://srtm.csi.cgiar.org/srtmdata/). Aerial photography (**C**) was generated using Google Earth Pro software (v7.3.4.8642 https://www.google.com/intl/es/earth/).

El Espinoso is located on the central coast of the Cantabrian Region (northern Spain), in the easternmost sector of Asturias province (Fig. 1), which is characterized by an Atlantic climate. The cave opens out from a 20-m-high limestone cliff, which dominates a closed valley, at only 200 m from the present Atlantic shoreline. The cave entrance is oriented towards SW, and to access it is necessary to climb a four-meter-high part of the cliff. At the end of the cavity, a collective burial was discovered, where the human bones were scattered around the cave floor, highly fragmented and with neither anatomical connections nor associated grave goods. Data from three humans recovered from the regional sites of La Llana, Los Cinchos and La Fragua caves, belonging to the Early and Middle Bronze Age, were used as a comparison with those found at El Espinoso (See Supplementary Texts 1 to 4 for more information about these sites). Recently, provisional genetic results from El Espinoso’s individuals have shown a Steppe ancestry of almost 30%^36^, constituting one of the Bronze Age sites with the greatest proportion of this ancestry in Iberia. Genetic evidence demonstrates that human migrations introduced the Steppe ancestry in Iberia during the Chalcolithic/Bronze Age transition^37^. This trend was remarkably stronger in northern Iberia, giving clues by the place where these populations arrived. The incoming ancestry is even more significant in El Espinoso, suggesting a possible second flow of people in the Late Bronze Age. These population dynamics may have caused social and economic changes in northern Iberia, including the potential arrival of new crops.

## Results

### Radiocarbon dating and Bayesian modelling

**Figure 2:**
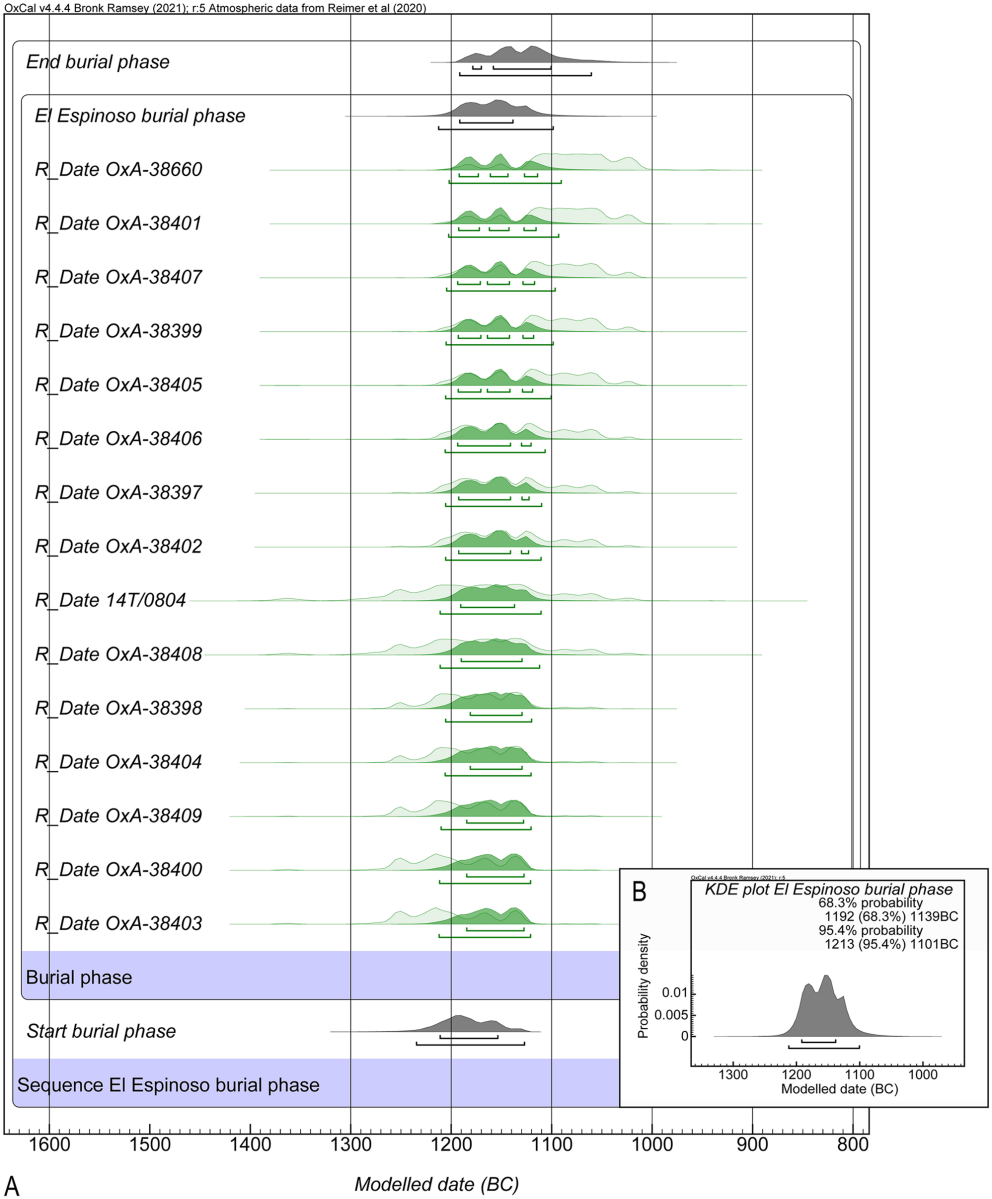
(**A**) Radiocarbon dates from El Espinoso cave modelled in OxCal v4.4.2 against IntCal20 curve. (**B**) KDE plot of the likelihood distributions of El Espinoso burial phase.

Fifteen individuals of the total of 20 were directly dated. All the dates fall in the last third of the second millennium cal. BC, corresponding to the Late Bronze Age (Supplementary Table 1)^38^. The El Espinoso sequence showed a convergence greater than 99%, and the model and overall agreement indices were 107.9% and 100.2%, respectively. Only OxA-38660 date had a poor agreement of 52.9%. A model of the burial phase at the site was built with start and end boundaries (Fig. 2). This model was replicated up to three times to prove its reliability, and no more outliers were found. The model provides a start date between 1235 and 1129 cal. BC and end date between 1214 and 1099 cal. BC (at 95.4% probability). Thus, the formation of the assemblage spans between 1235 and 1099 cal. BC and the maximum duration of the burial phase is 115 years. KDE plot shows three events along the funerary sequence of the site with greater importance of the central peak when the summed probability distribution suggests that most individuals were buried. Thus, the El Espinoso cave was used for funerary practices for a relatively short time (a few generations, at most). These data constitute a well-defined, almost synchronic event when the deceased individuals of a specific population were repeatedly introduced into the cave.

### Stable isotope analysis

**Figure 3:**
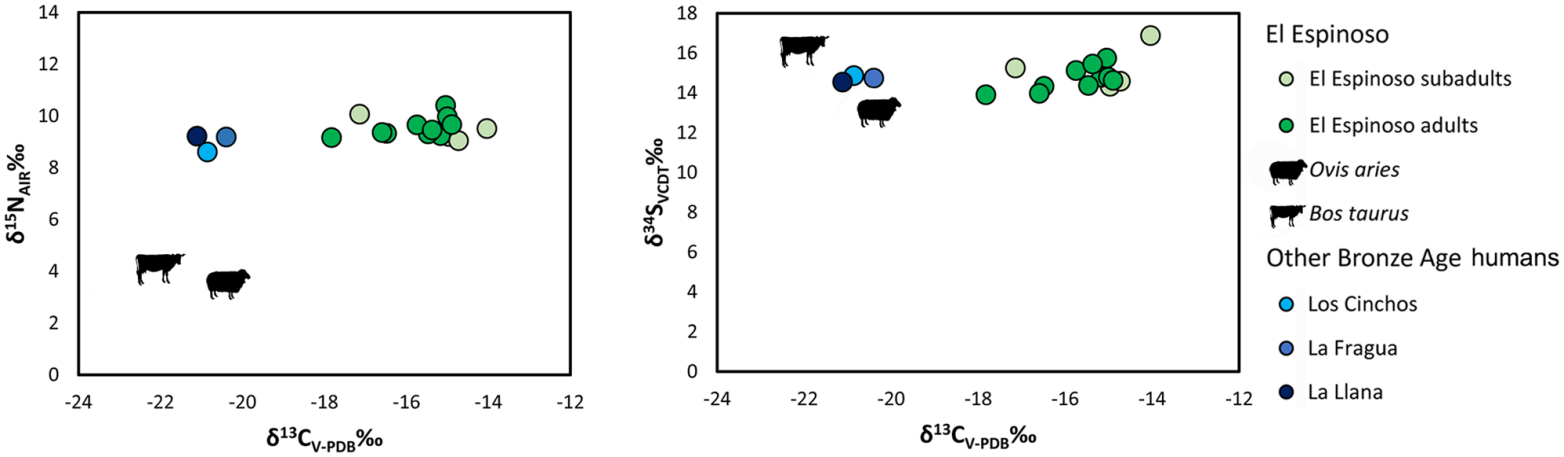
Scatterplot of human and animal bone collagen δ^13^C, δ^15^N and δ^34^S values of El Espinoso cave and δ^13^C, δ^15^N and δ^34^S values of human bone collagen from La Llana, Los Cinchos, and La Fragua caves.

The stable isotope values for the human and animal remains are reported in Supplementary Table 2 and plotted in Fig. 3. Collagen extraction was successfully undertaken in all the samples with %Col > 5. Their %C, %N, %S, C:N, C:S, and N:S elemental ratio values indicate good collagen quality^39–42^.

From El Espinoso cave, the humans analyzed (*n* = 14) had δ^13^C values ranging between − 17.8 and − 14‰ (*m* = − 15.6‰), δ^15^N values between 9.1 and 10.4‰ (*m* = 9.5‰) and δ^34^S values between 13.5 and 16.9‰ (*m* = 14.9‰).The two faunal specimens available were analyzed as a local baseline from the site: a cow specimen reported a δ^13^C signature of − 22.1‰, a δ^15^N value of 4.1‰ and a δ^34^S signature of 16.2‰, and a sheep had a δ^13^C value of − 20.6‰, a δ^15^N signature of 3.7‰ and a δ^34^S value of 13.5‰. Humans from La Llana, Los Cinchos, and La Fragua sites reported δ^13^C values of − 20.9‰, − 21.1‰ and − 20.4‰ and δ^15^N values of 8.6‰, 9.2‰ and 9.2‰ and δ^34^S values of 14.9‰, 14.5‰ and 14.8‰, respectively.

### Dental calculus analysis

Seventy-four starch grains were found in the dental calculus of 16 individuals (Supplementary Table 3). Based on their morphometric characteristics, two different types of grains were identified and assigned to the Triticeae and Paniceae tribes of the Poaceae family (grasses). Both morphotypes were documented in suitable proportions. Despite the small size of the calculus samples, most of the starch grains had an excellent preservation state. Microremains, like the ones identified in the analyzed dental calculus, were not retrieved in the dust traps placed in the laboratory, supporting the archaeological nature of the microfossils identified in the calculus samples. Sediment samples were analyzed as well and showed a great diversity of microremains, including phytoliths and fungal spores, much more abundant than in the dental calculus samples. Starch grains were not recovered from sediment samples.

#### Morphotype I

**Figure 4:**
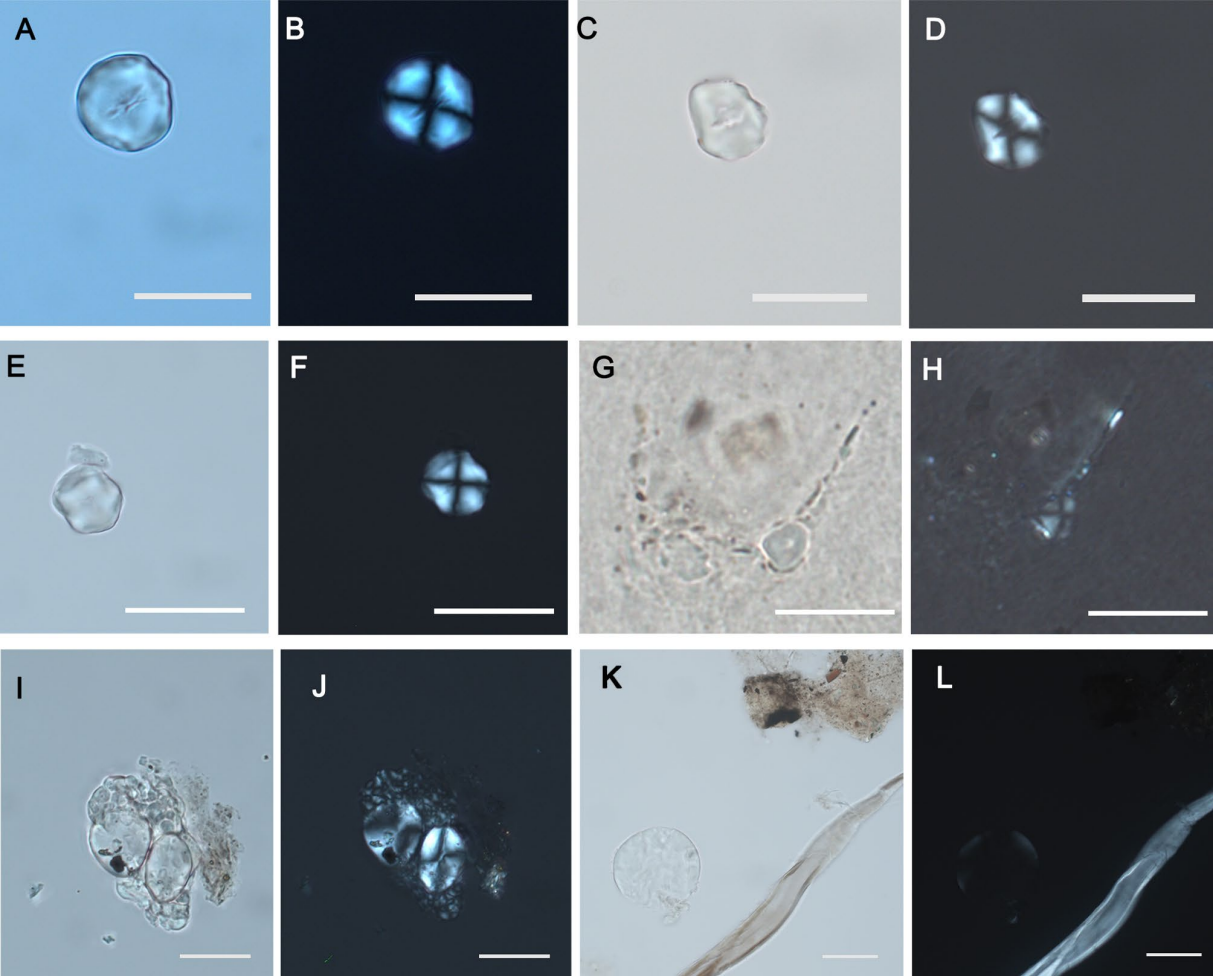
(**A**–**L**) Starch grains embedded in El Espinoso's dental calculus in light microscope and polarised light microscope. (**A**–**H**) Polyhedral starch grains with central hilum and fissures associated with the Paniceae tribe; (**I**–**L**) Round to oval starch grains "bimodal distribution" consistent with the Triticeae tribe. (Scale bar, 20 μm).

The first morphotype of starch grains represents 60.8% of the total, and it is characterized by a bimodal distribution typical of most grasses of the Triticeae tribe (Supplementary Table 3) (Fig. 4E–H). Such distribution is equally frequent in the samples. Large grains (≤ 15 μm), defined as type A, are round to oval in 2D shape, lenticular in 3D, with central or slightly sunken hilum and few lamellae. The largest grains are similar to those of cereals like wheat and barley. Smaller grains (< 10 μm), defined as type B, are almost spherical and have a central hilum^43,44^. They potentially also belong to the Poaceae family, although similar grains can be found in many other plants. The mean dimension of these starch grains is 13.8 μm.

#### Morphotype II

39.2% of the starch grains come from this morphotype. They have a 3D polyhedral shape, with a central hilum and fissures radiating from it. The extinction cross is visible, with straight arms, while lamellae are not identified (Supplementary Table 3) (Fig. 4A–D). This type of grain is abundant in the samples and is consistent with those of the Paniceae tribe, likely representing millets. The mean dimension of these starch grains is 14.5 μm.

A variety of other plant structures were found as well (Supplementary Fig. 8). These microremains of probable non-dietary origin include fungal spores, charcoals, fibres, wooden tissues, and several conifer pollen granules.

## Discussion

### Stable isotope evidence of millet consumption

**Figure 5:**
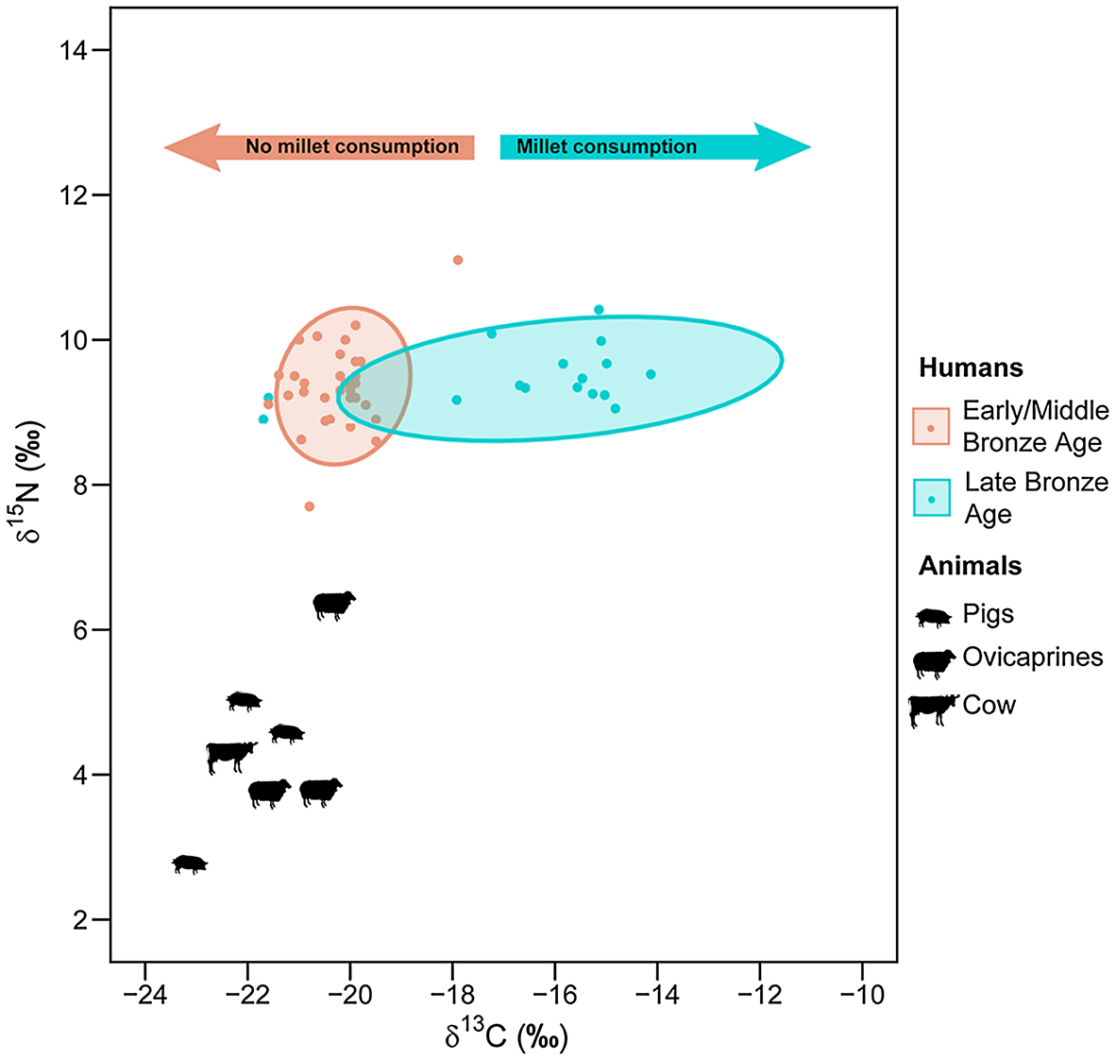
δ^13^C and δ^15^N values of bone collagen analysed from northern Atlantic Iberian Bronze Age sites, including humans and animals from El Espinoso (Late Bronze Age); and La Llana, Los Cinchos and La Fragua (Early/Middle Bronze Age) in this study. Cova do Santo, La Castañera, La Garma A, Santimamiñe (Early/Middle Bronze Age) and Ondarre (Late Bronze Age) from previously published data.

The El Espinoso animal samples had δ^15^N signatures 3–4‰ lower than the human ones, which constitutes the expected relationship between consumers and prey^45,46^. Nitrogen isotopes from other Bronze Age contexts in northern Iberia, such as Cova do Santo^47^ and La Castañera^48^, show similar trends between humans and animals (Fig. 5). The animals had δ^13^C values typical for a terrestrial European C_3_ ecosystem^49^, while the carbon results from El Espinoso human individuals showed that they were significantly enriched in ^13^C, suggesting an intake of C_4_ plants or marine foods^50,51^. As the length of the aquatic food chain is longer than the terrestrial one, nitrogen isotope values can be used to discard the hypothesis that the individuals ate marine foods, which would reflect higher nitrogen values, especially fish or mammals^46^, which would be reflected in higher δ^15^N values. Shellfish can overlap signals with C_4_ plants, but despite the proximity of the site to the sea, there is no archaeological evidence of marine resource consumption (shells or fish remains) during the regional Bronze Age, neither in the cave deposits nor in the few documented open-air sites^38,52^. However, future archaeomalacological and ichthyofaunal studies will help to verify this.

In addition, to reject the possible influence of fish on the El Espinoso human diet due to the higher δ^13^C values, sulfur isotope ratios were also analyzed. The freshwater and marine resources consumption produce elevated δ^34^S values^53^. At El Espinoso, humans but also faunal specimens, who follow different diets, showed the dispersion of the same values ranging between 13.5 and 16.9‰. On the other hand, La Llana, La Fragua and Los Cinchos individuals reflected similar sulfur values, but they did not provide enrichment of carbon values. Therefore, the δ^34^S values recorded were more likely to be associated with the environment where they were living rather than with the influence of fish in the diet. Indeed, humans and animals of El Espinoso were predominantly living near the coast, where the sea spray effect can reach more than 30 km inland, affecting the sulfur signatures of coastal environments^54^. In this sense, the sulfur isotope values from humans and animals of Los Avellanos I and II, dated in the Late Neolithic/Chalcolithic and without typical carbon isotope values of marine diets, showed the same higher sulfur isotope signals^55^. Consequently, the most likely hypothesis to explain the δ^13^C enrichment in El Espinoso individuals could be a significant consumption of C_4_ plants, likely millets. Isotopic measurements of modern millets have provided δ^13^C values from − 10 to − 12‰ and δ^15^N values from 3 to 4‰^56^, as well as isotopic measurements of Iron Age millets have reported δ^13^C and δ^15^N values around − 10‰ and 4‰^57^. Millet has a protein content of approximately 10%; thus, a significant intake of these crops is required to detect a substantial change in collagen isotope ratios^58^.

In contrast to El Espinoso, the isotopic data from the humans of La Llana, Los Cinchos, and La Fragua sites reported lower δ^13^C values, showing that the diet was predominantly terrestrial, based on C_3_ plants and animal protein. This dietary pattern was also observed in other human bone collagen isotopic studies from the Bronze Age sites, such as Cova do Santo^47^, La Castañera^48^, La Garma A^59^, Santimamiñe^60^ and Ondarre^61^ (Fig. 5). These archaeological sites are dated to the Early and Middle Bronze Age, except for one individual from Ondarre, which belongs to the Late Bronze Age. Still, surprisingly, it showed an opposite carbon value to the El Espinoso group. In all of the groups mentioned above, isotopic signals of C_4_ plant consumption were not detected, which suggests that millets were probably not eaten yet in northern Iberia during these periods. In this sense, dentine sequential analysis of carbon and nitrogen isotopes from seven human teeth documented in Los Canes cave (Asturias), dated in the Late Bronze Age, showed isotopic signals of C_4_ plant consumption during the childhood, while two more teeth dated in the Early Bronze Age indicated a C_3_ exclusive diet^62^. With the current isotopic evidence, if C_4_ plant resources were consumed before the Late Bronze Age, the quantities were not high enough to be registered in the long-term bone collagen record. This would suggest infrequent or occasional cultivation of millets during the Middle Bronze Age, becoming a staple crop only in later periods, probably from Late Bronze Age, as the isotopic results from El Espinoso cave demonstrate. Until now, the earliest bone collagen isotopic evidence of C_4_ plant consumption in northern Iberia is documented at La Hoya village, an inland site 150 km from the current Atlantic sea in Alava and dated in the Iron Age^37^. El Espinoso individuals are more than 700 years older than La Hoya, setting back the isotopic evidence of intensive millet consumption in northern Iberia to the Late Bronze Age.

According to the evidence, millets were introduced into Iberia across the Pyrenees from Central Europe during the Middle/Late Bronze Age transition, reaching earlier and playing a more important role in the northern regions of Iberia than the southern ones^63^. A longitudinal gradient for the dispersal from Eastern to Western Europe during recent prehistory is proposed, with the Atlantic coast being one of the last regions to adopt millets. However, the radiocarbon evidence indicates a relatively quick spread of these crops through Europe^20^. Concerning the published stable isotope analyses from different European regions, some studies have found isotopic evidence of millet consumption around 5500 cal. BC in East Asia^64,65^, between 2500 and 2000 cal. BC in Central Asia^17,66^, between 1600 and 1400 cal. BC in the Caucasus region^67^, between 1600 and 1200 cal. BC in northern and central Italy^68,69^, between 1400 and 1200 cal. BC in France^70^ and between 1300 and 1000 cal. BC in northern Spain.

Regarding the carbon values across the humans’ age distribution of El Espinoso, three out of four subadults showed the higher carbon values of the sample, suggesting the existence of inequalities in C_4_ plant consumption by age. These minor differences can be explained by the higher importance of millet in the children's diet. This pattern was previously observed at La Hoya and Los Canes^62,71^. Here millets were introduced to infants and young children during the weaning process, probably in the form of porridge^57^. Considering the stable isotope data from different millet consumers of Europe, in some cases, all the individuals of the same group ate large amounts of millet, while in others, the millet consumers lived in a community where most people did not consume this crop. This fact suggests dietary differences related to social, cultural, or economic status^33^. However, the current isotopic evidence indicates that intensive consumption of millets outside China was sporadic during recent prehistory until the first millennium cal. BC, when the results tend to be more homogeneous from the Eurasian steppes to the Atlantic fringe.

### Dental calculus evidence of millet consumption

The richest archaeological evidence of millet grains within dental calculus comes from Asia, where these crops were primarily domesticated. Different studies have revealed starch grains and phytoliths from other millet species^72–76^. Based on the phytolith and starch grain morphology, these crops show great potential for identifying different genera or species, opening new possibilities for their detection in archaeological contexts^31^. There is little evidence of preserved millet grains in dental calculus from Europe, with even fewer examples in Iberia. This lack of knowledge can be explained by the absence of studies on the recent prehistory of this geographical area. Among a great diversity of taxa represented, some starches of the Paniceae tribe were recovered within the dental calculus of the Chalcolithic individuals from El Mirador cave (Burgos, Spain), based on their average size and morphological features^77^. In Italy, 79 starch grains were identified among individuals from the Chalcolithic/Bronze Age site of Grotta dello Scoglietto (Tuscany)^28^.

Polyhedral starch grains (Morphotype II) found in the El Espinoso individuals may be attributed to different C_4_ plants. Experimental starch grains from *Panicum miliaceum* (Supplementary Fig. 5) or *Setaria italica* (Supplementary Fig. 6) conducted in this work share these sizes and morphological features, although slight differences can be identified in the size classes and fissure patterns. *Setaria italica* can have occasional oval grains and a centric hilum traversed by fissures which vary in form. In contrast, starch grains from *Panicum miliaceum* have a mostly polyhedral shape with a centric hilum where fissures are less common^32^. Experimental starch grains size differs slightly between both species, being marginally smaller than the grains of *Panicum miliaceum*. Wild millet species, such as *Setaria viridis* or *Setaria verticillata* are also smaller, with a more characteristic spherical morphology and short fissures^31^ (Supplementary Table 4) (Supplementary Fig. 9). The morphological features and the mean dimensions of the archaeological starch grains suggest that *Setaria italica* was the crop consumed by this human group. However, both species might have been consumed in a mixed way since millet polycropping has been suggested archaeologically and documented ethnographically^7,63^. The presence of millet starches in El Espinoso individuals, although in small quantities, can be considered conclusive, confirming the millet consumption suggested by the stable isotope values. However, phytoliths typical of millet species have not been documented. The low presence of starch grains and the absence of phytoliths could be explained by the small size of the calculus samples. Only small amounts of dental calculus could be sampled from most individuals, even less than 1 mg (Supplementary Table 3). This can be considered satisfactory since it has allowed microfossils extraction in such small quantities, establishing size limits for further studies.

Starch grains with an oval morphology and "bimodal distribution" (Morphotype I), typical of grass grains, as those of the Triticeae tribe (Supplementary Fig. 7), were also identified in reasonable quantities as millet starch grains. Previous experimental analyses in species of the Triticeae tribe have revealed that wild starch grains (Aegilops *genus*) show a larger size distribution than domestic ones (Triticum and Hordeum *genus*)^78,79^. Additionally, B-type grains are more abundant than A-type in wheat and barley species. Our experimental measurements have shown the great variety of starch grain sizes for the different Triticeae domestic species (Supplementary Table 4) (Supplementary Fig. 9). However, the morphology and size obtained for the archaeological Morphotype I starch grains are consistent with the wheat and barley species. This evidence complements the isotopic data that indicates the great importance of millet in the human diet at El Espinoso and the relevance of other C_3_ plants. As the millets allowed for two growing seasons per year, other crops such as wheat or barley could also have been cultivated along with them annually. These findings reveal diversified agriculture during this time, with the millet being complemented by meat, dairy products, and other cereals. Concerning the other plant residues entrapped within the tartar, they may have been related to a wide range of non-dietary activities such as oral hygiene with toothpicks, the para-masticatory use of the mouth in various tasks, inhalation of airborne paleoenvironmental debris due to the exposure to hearths, and craft activities such as cordage, textile, basketry or net-making^80^.

### Macro-botanical evidence of millets in the archaeological record of northern Iberia

Millets are poorly represented in the Bronze Age archaeological record. The first carpological evidence of millet exploitation in Iberia dates to the Middle Bronze Age. However, they were not systematically exploited until the Late Bronze Age and Iron Age^1^. No carpological evidence of the wild native species that are relatives of the domesticated millets (*Panicum repens, Setaria viridis,* or *Setaria verticillata*), also known as “forgotten millets”, has been found during the recent prehistory of northern Iberia^81^*.* Today they are widely distributed across central and south Europe^82^ but almost unknown in Atlantic Europe^83^. The only C_4_ plants found in Iberia during recent prehistory are domestic millets, particularly broomcorn and foxtail millets (*Panicum miliaceum* and *Setaria italica)*. They are the C_4_ crops that are frequently identified across Europe^33^, constituting the primary candidates cultivated in large proportions by northern Iberian farmers. In the Vizcaya province, macroremains of foxtail millet were discovered in Kobaederra (Level 1) and Arenaza (Layer 9), chronologically assigned to the Chalcolithic and Early Bronze Age, respectively^84^. However, those seeds have not been directly dated; thus, we cannot exclude the possibility that they might be intrusive. Alternatively, there is an absence of *Panicum milliaceum* in the prehistoric carpological records of the Cantabrian Region. Also, in northern Portugal, several millet grains were found in the Chalcolithic sequence of Crasto de Palheiros^85^, but stratigraphic issues were reported in those layers. In the Middle Bronze Age in northern Portugal, the carpological remains of broomcorn millet were identified in the sites of Sola IIb^86^ and Terraço das Laranjeiras^87^. Later, during the Late Bronze Age, a higher presence of millet (exclusively *P. miliaceum*) is recorded across the region, in the neighboring areas of Galicia province (A Fontela, Penalba, and Penarrubia) and northern Portugal (Castelo de Matos, São Julião, Santinha, and Senhora da Guia)^9^. Likewise, seeds from both millet species have been identified in different sites in the Ebro basin (northern Mediterranean Iberia) during the Middle and Late Bronze Age, such as at Punta Farisa, Masada de Ratón, El Vilot and Vincament^88,89^.

Consequently, the available evidence confirms that millet cultivation in northern Iberia seems to have started during the Middle/Late Bronze Age transition. However, it was not until the Late Bronze Age that millets became a significant and regular crop. The transition from the Middle to Late Bronze Age marks the shift of millets from a minor to a major role in the agriculture of the European farming societies^90^. To better understand the origin and development of millet cultivation, direct dating of millet grains will be needed^20^ as the morphology and size of these tiny seeds facilitate their vertical movements downwards through the stratigraphical sequences and must be called into question for future research^23^.

### Ethnographic insights of millets in northern Spain

Ethnographic studies can provide valuable information that can be used as a complement to the archaeological evidence about the cultivation and consumption of millets in the past. This is the case in northern Spain, where *Setaria italica* (foxtail millet) and *Panicum miliaceum* (broomcorn millet) were traditionally cultivated. Ethnobotanical data have shown a higher presence of *S. italica* or both species sown together in the Asturias province. Such place-names as "Panizales, Panicera, Paniceres," etc., are familiar in the Asturias and refer to the Spanish name of foxtail millet^91^. At the same time, the most common crop found in Galicia and northern Portugal is *P. miliaceum*^7^. This coincides with the archaeobotanical remains recovered from prehistoric sites, with foxtail millet found in the Cantabrian Region and the broomcorn millet in the northwestern area of Iberia. Ethnographic approaches in Galicia during the twentieth century have provided insights into the utilization of millet grains for human consumption. Simultaneously, both grains and plants are used as feed or fodder for animals^35^. In recent times in the wider northwestern regions of Iberia, millet has been used to make porridge, stews, puddings, or bread for human consumption^7^. However, there are some differences observed among regions. In Asturias, *S. italica* is used exclusively for foddering, while in Galicia and northern Portugal, besides animal consumption, *P. miliaceum,* is also used as human food. Both crops have been cultivated until the present, and their importance was exceptionally high during the Roman and Medieval periods, as testified by ancient sources^92^ and stable isotope analyses^93^. However, the arrival of maize (*Zea mays*) from America in the seventeenth century displaced their cultivation to a marginal role. Also, ethnographic studies in other European regions reported the use of millets to produce an alcoholic drink^94^.

## Conclusions

In this study, we infer direct and precise data about the systematic use of millets during the Late Bronze Age in northern Iberia by combining different methodologies approaches on human remains including: (1) radiocarbon dating; (2) stable isotope (δ^13^C, δ^15^N and δ^34^S); and (3) dental calculus analysis. El Espinoso cave was used as a burial location during the last third of the second millennium cal. BC. The Bayesian model places the span of the burial phase between 0 and 115 years, supporting a practically synchronous use of the cave for a few decades. δ^13^C and δ^15^N isotope values show a diet with high consumption of C_4_ plants, likely millets, and contrast with the values obtained in different Early and Middle Bronze Age funerary sites for the same region, suggesting that the systematic consumption of millets in this area started from the Late Bronze Age. The study of the microresidues entrapped within the dental calculus of El Espinoso individuals confirms the presence of starch grains with a polyhedral morphology typical of the Paniceae tribe. Besides, other round to oval starch grains characteristic of the Triticeae tribe have also been identified, demonstrating a mixed agricultural economy for the Late Bronze Age of northern Iberia. The multidisciplinary research undertaken on the human bone assemblage of El Espinoso cave has generated a corpus of high-quality biomolecular data about millet consumption and constitutes one of the oldest and most direct evidence of the exploitation of millets in northern Atlantic Iberia.

## Methods

In this research, a multidisciplinary approach was conducted to reassess the relevance of millets in the prehistoric human diet. Radiocarbon dating was carried out to establish a precise chronology for the humans analyzed here. The dates were calibrated in OxCal v4.4^95^ using the IntCal20 calibration curve^96^. All results are presented at a 95.4% probability. The results were modelled using Bayesian statistics. Stable isotope analyses (δ^13^C, δ^15^N, δ^34^S) of humans and animals were performed to ascertain these individuals' carbon, nitrogen, and sulfur signatures. Bone collagen extraction was conducted in the EvoAdapta Group (University of Cantabria) according to the procedures proposed by Richards and Hedges^97^. Carbon, nitrogen and sulfur isotope analysis of the collagen samples was undertaken by Elemental Analysis—Isotope Ratio Mass Spectrometry (EA-IRMS) in Iso-analytical laboratory (Crewe, UK). Quality indicators widely used were employed^39–42^. Dental calculus analysis was achieved to discern what types of plants were involved in the diet of this human group. The dental calculus matrix was removed from the teeth following the protocol by Sabin and Fellow-Yates^98^. Decontamination and the extraction procedures for micro-debris were carried out following standard protocols as described by Cristiani et al.^43,99^ and Fiorin et al.^100^, and were conducted in dedicated clean spaces under strict environmental monitoring of the DANTE—Diet and ANcient TEchnology laboratory of Sapienza University of Rome (Italy). The analysis of the microfossils was carried out using a Zeiss Imager2 polarised microscope (100×–630×) at the DANTE laboratory and a Leica DVM6 M digital microscope at the EvoAdapta Group. Morphological and size comparisons were carried out with the modern plant reference collections housed at these institutions (See Supplementary Text 5 for more detailed descriptions of methods).

## Supplementary Information

Supplementary Information.

## Data Availability

All the data reported in this article are provided in the Figures and Tables of the Supplementary Information and Manuscript files. Radiocarbon dates are listed in the Supplementary Table 1, as well as the Bayesian modelling which are included in Fig. 2. Stable isotope analyses on bone collagen (δ^13^C, δ^15^N and δ^34^S) and the quality indicators of the samples were provided in Supplementary Table 2 and plotted in Fig. 3. The archaeological microremains extracted from the dental calculus are described in Supplementary Table 3 and shown in Fig. 4. and Supplementary Fig. 8. Experimental starch grains from the species involved in this study are included in Supplementary Figs. 5, 6 and 7. Statistical analysis of the length measurement of these starch grains is reported in Supplementary Table 4 and Supplementary Fig. 9. All the other chronological and regional data supporting the findings and interpretations of this study are available in existing publications referenced in the text and Supplementary Information. The archaeological remains studied and sampled in this research are curated in the laboratory of EvoAdapta Group (University of Cantabria, Spain). These materials will be returned to Museo Arqueológico de Asturias (Oviedo, Spain) and Museo de Prehistoria y Arqueología de Cantabria (Santander, Spain).
