# Supplementary material for: The arrival of millets to the Atlantic coast of northern Iberia
Source: Sci Rep. 2022 Nov 3;12:18589. doi: 10.1038/s41598-022-23227-4 (PMC9633756; doi:10.1038/s41598-022-23227-4)
Supplement: Supplementary file 1 — Supplementary Information. [file 41598_2022_23227_MOESM1_ESM.pdf]

Supplementary Information for

The arrival of millets to the Atlantic coast of northern Iberia

Borja González-Rabanal[1,*], Ana B. Marín-Arroyo[1,*], Emanuela Cristiani[2], Andrea Zupancich[2,3], M. R. González Morales[4]

[1]Grupo de I+D+i EVOADAPTA (Evolución Humana y Adaptaciones durante la Prehistoria). Dpto. Ciencias Históricas. Universidad de Cantabria. Av. Los Castros 44, 39005 Santander, Spain
[2]DANTE - Diet and ANcient TEchnology Laboratory, Department of Oral and Maxillo-Facial Sciences, Sapienza University of Rome, Via Caserta 6, Rome 00161, Italy
[3]Archaeology of Social Dynamics, Institución Milá y Fontanals, Spanish National Research Council (CSIC), Barcelona, Spain
[4]Instituto Internacional de Investigaciones Prehistóricas de Cantabria (IIIPC), Universidad de Cantabria - Gobierno de Cantabria. Santander, Spain

*Corresponding author: borjagrabanal@gmail.com; anabelen.marin@unican.es

This PDF file includes:

Supplementary Text 1: El Espinoso cave
Supplementary Text 2: La Llana cave
Supplementary Text 3: Los Cinchos cave
Supplementary Text 4: La Fragua cave
Supplementary Text 5: Methods
Supplementary Figures 1 to 9
Supplementary Tables 1 to 4
Supplementary References

Supplementary Text 1: El Espinoso cave

Excavations at El Espinoso were carried out in the early 1980s when an anthropic deposit pertaining to the Upper Palaeolithic was discovered in the cave vestibule[1]. Radiocarbon dating and the lithic industry recovered in a reduced test pit placed this occupation in the Archaic Magdalenian period[2]. In 1993, abundant human remains, documented across the surface of the cave floor in a ~40m^2 chamber located at the end of the cavity, were recovered for study (Supplementary Fig. 1). The anthropological study of the human remains has demonstrated that the cave was used as a collective burial place to deposit individuals of different ages and sexes. All together 1,230 human remains belonging to 20 individuals were identified: two infants (0-3 years), four children (3-12 years), five adolescents (12-20 years), seven adults (20-50 years) and two old adults (over 50 years). Sexual identification was only possible for twelve individuals: seven males and five females. Stature estimation gave an average height of 1.71 m for adult males and 1.60 m for females. Some pathologies were found, including caries, dental calculus, periodontal disease, osteoarthritis and antemortem fractures[3].

Taphonomic analysis of the assemblage showed poor anatomical representation in the collection and its high degree of fragmentation. There is an important representation of short bones and low-density bones, such as carpal and tarsal, which indicates the primary character of the burials. However, skulls and long bones are poorly represented, and when present, they are widely fragmented. Therefore, an anthropic origin for the differential preservation within the deposit cannot be excluded. This could be related to the extraction of the most diagnostic bones from the cave, once the bodies were decomposed, to carry out a secondary deposition somewhere else, leaving in the cave many of the smaller bones. Taphonomic processes reported significant post-depositional diagenetic activity related to water circulation and the humidity typical of surface deposits in karstic environments[4].

A tooth from this funerary deposit was dated to the second half of the second millennium cal. BC (ICA-14T/0804: 2960 ± 40 BP; 1369-1019 cal. BC), corresponding to the local Late Bronze Age[5].

Supplementary Text 2: La Llana cave

Another human individual studied here came from La Llana cave also in coastal Asturias, Spain. The archaeological site was discovered in 1981 during an archaeological survey[6] and was later excavated by M. R. González Morales between 1982–1985[1]. The cave has two entrances: a large mouth located in a deep doline at its northern end and a small rockshelter to the south (now almost fully obstructed by anthropic sediments). The archaeological excavation inside the cave was divided into two different areas: Sector A, where a human burial was found, and Sector B, where a Mesolithic shell midden was identified. The skeleton was lying on the surface, with some bones covered by a stalagmitic flowstone firmly cementing them to the cave floor (Supplementary Fig. 2). In 1983, the human bones were gently cleaned with a soft brush and water and photographed in situ. The remains that were less adhered to the floor or at risk of being damaged were recovered to prevent their destruction. This was the case for the cranium, whose left side was broken *postmortem* and had sunken into the cranial vault. The skeleton belongs to an adult male with a stature of 165-168 cm. This man suffered a perimortem otitis media[7]. A taphonomic study of the human remains shows that some bones were still articulated, whereas other bones had been displaced due to the decomposition of flesh and a low-intensity water flow responsible for the calcite layer covering the bones. A coxal fragment was dated to the Middle Bronze Age (UGAMS-9083: 3300 ± 25 BP; 1618-1510 cal. BC)[8].

Supplementary Text 3: Los Cinchos cave

The human skeleton from Los Cinchos cave was located in a cavity 1870 meters above sea level in the Natural Park of Las Ubiñas (Asturias). The skeleton was found in 2010 at the bottom of a complex karstic system composed of four different pits[9]. The human remains were located in a narrow crack in the bedrock and deposited on an irregular platform of debris clogging the crevace (Supplementary Fig. 3). The skeleton had been placed in a sitting position, although it did not

maintain its anatomical disposition due to postdepositional movements caused by the decomposition of the corpse and postmortem manipulations of the bones. No structures, ornamental elements, or grave goods were documented. The anthropological study determined that it was an 18-19-year-old male juvenile with a height of 166 ± 6.9 cm. Even though the dental eruption had ended, the individual presented a considerable delay in the fusion of the epiphyses, with some epiphyses fused or in the process of fusion, and others completely unfused, which has been linked to congenital disease. On the other hand, the paleopathological study ruled out any type of perimortem injury because of an accident[10]. The interpretation of the find is that it was related to the voluntary or forced descent of the individual with the help of other people into the pit, from it was impossible to exit. A bone from this individual was dated to the Early Bronze Age (Beta-366545: 3550 ± 30 BP; 2014-1771 cal. BC)[9].

Supplementary Text 4: La Fragua cave

La Fragua site is a relatively small cave located at 125 meters above sea level on the south-eastern slope of Mount Buciero, on the shore in Santoña (Cantabria). Excavations from 1991 to 1996 provided a well-defined stratigraphic sequence. Archaeological levels of the site consist of the following: Level 4 (Magdalenian), Level 3 (Azilien), Level 2 (sterile archaeologically) and Level 1 (Mesolithic)[11]. During the excavations a deposit of mixed fill, limestone blocks, pottery, animal and human bones was identified inside a trench that had completely cut through the stratigraphic sequence of the cave and that was filled in part with material from Level 1 (Supplementary Fig. 4). The trench was sealed by hearths made by shepherds who used the cave in the last century. The trench was dug subsequently to the deposition of Level 1, but its absolute chronology is unknown. Seven human remains were identified in the fill of the trench: a fragment of the right humerus, a left coxal bone, three hand phalanges, a right third cuneiform and a decidual tooth. They belong to an MNI of two (one adult and one infant). However, the presence of a second individual with only a decidual tooth is unverifiable. No diagnostic bones like cranium, mandible or long bones have been documented. Therefore, the human record is composed of small and low-density remains, bones that generally are not preserved in secondary burials but are found in primary burials. 140 sherds of vessels and 517 remains of both domesticate and wild faunal bones were identified inside the trench[12]. At the bottom of the trench appeared a slab within a small ditch. This slab could have constituted a marker for the burial. This set of evidence could indicate an opening of the trench to remove the main human bones of the skeleton to give them a secondary burial elsewhere. A radiocarbon date from a human humerus offers a chronology referring to the Early Bronze Age (OxA-31057: 3697 ± 30 BP; 2199-1978 cal. BC)[13].

Supplementary Text 5: Methods

Radiocarbon dating. C^{14} dating programme of the collective burial at El Espinoso cave was conducted to identify the funerary episodes at the site. Fourteen new human individuals from the total of 20 were selected for AMS dates. Given the scrambled character of the funerary context and to avoid resampling of the same skeleton, the selection of the bones was based on the Minimum Number of Individuals (MNI) considering the side, size and age of the most represented bone. Two additional criteria were added for the sampling: priority for long bones taxonomically and anatomically identifiable and well-preserved remains to ensure a high-quality standard of collagen preservation. In this case, the best represented long bone was the ulna. The dates were calibrated in OxCal v4.4 using the IntCal20 calibration curve[14]. All results are presented at a 95.4% probability. The results were modelled using Bayesian statistics[15]. The Bayesian model was built with a *Phase* and *Boundary* functions to identify the beginning and end of the El Espinoso burial phase. A t-type outlier model was adopted with an initial 5% probability for each determination to be an outlier. An agreement of 60% or higher is considered an acceptable result. A *Date* function was used to estimate the duration of the burial phase. Finally, a *KDE plot* function was generated to summarize the likelihood distributions of funerary events within the site[16].

Stable isotope analyses. Bone collagen was extracted on 19 samples to obtain carbon, nitrogen and sulphur isotopic values. Samples represent the 14 newly dated humans and two additional animals from El Espinoso cave. Unfortunately, no other faunal remains were available within the burial deposit. Three additional human individuals from the Early and Middle Bronze Age Cantabrian sites of La Llana, Los Cinchos and La Fragua caves were sampled to compare with those of El Espinoso. Sample preparation was undertaken using facilities of the EvoAdapta Group (University of Cantabria), where bone collagen extraction was undertaken according to the procedures proposed by Richards and Hedges[17]. This method involves the following steps: 1) cleaning of the bone fragments (0.6-0.8 g) by abrasion to remove any possible contamination; 2) demineralization of the samples in 0.5 M HCl at 6-8 °C for 3-10 days; 3) washing using de-ionized water; 4) gelatinization of the samples in a weak solution of pH 3 HCL at 70 °C for 48 h; 5) filtration with 5-8 μm Ezee® filters; 6) freeze-drying of the samples.
Samples were analyzed for $\delta^{13}C$, $\delta^{15}N$ and $\delta^{34}S$ using a Europa Scientific™ elemental analyzer, coupled to a mass spectrometer at Iso-Analytical laboratory (Crewe, UK). The $\delta^{13}C$, $\delta^{15}N$ and $\delta^{34}S$ values were reported relative to the V-PDB, AIR and VCDT international standards, respectively. The reference material used for carbon and nitrogen isotope analysis of the collagen samples was IA-R068 (soy protein, $\delta^{13}C$= -25.22‰, $\delta^{15}N$= 0.99‰). IA-R068, IA-R038 (L-alanine, $\delta^{13}C$= -24.99‰, $\delta^{15}N$= -0.65‰), IA-R069 (tuna protein, $\delta^{13}C$= -18.88‰, $\delta^{15}N$= 11.60‰) and a mixture of IAEA-C7 (oxalic acid, $\delta^{13}C$= -14.48‰) and IA-R046 (ammonium sulfate, $\delta^{15}N$= 22.04‰) were run as quality control check standards. IA-R068, IA-R038 and IA-R069 are calibrated against and traceable to IAEA-CH-6 (sucrose, $\delta^{13}C$= -10.45‰) and IAEA-N-1 (ammonium sulfate, $\delta^{15}N$= 0.40‰). IA-R046 is calibrated against and traceable to IAEA-N-1. IAEA-C7, IAEA-CH-6 and IAEA-N-1 are inter-laboratory comparison standards distributed by the International Atomic Energy Agency, Vienna. The reference material used for sulfur isotope analysis of the collagen samples was IA-R061 (barium sulfate, $\delta^{34}S$= 20.33‰). IA-R061, IA-R025 (barium sulfate, $\delta^{34}S$= 8.53‰) and IA-R026 (silver sulfide, $\delta^{34}S$= 3.96‰) were used for calibration and correction of the ^{18}O contribution to the SO^+ ion beam. IA-R061, IA-R025 and IA-R026 are in-house standards calibrated against and traceable to NBS-127 (barium sulfate, $\delta^{34}S$= 20.3‰) and IAEA-S-1 (silver sulfide, $\delta^{34}S$= -0.30‰). IA-R061, IAEA-S-1, IA-R068 (soy protein, $\delta^{34}S$= 5.25‰) and IA-R069 (tuna protein, $\delta^{34}S$= 18.91‰) were measured as quality control check standards during batch analysis of the collagen samples. IA-R068 and IA-R069 are in-house standards calibrated against and traceable to NBS-127 and IAEA-SO-5 (barium sulfate, $\delta^{34}S$= 0.50‰). NBS-127, IAEA-S-1 and IAEA-SO-5 are inter-laboratory comparison standards distributed by the International Atomic Energy Agency (IAEA) with internationally accepted $\delta^{34}S$ values. All quality controls standards provide homogenous results close to their consensus values and provide high precision measurements, with uncertainty lower than 0.1‰. One in every five samples were measured by duplicate and replication was typically <0.1‰ or better showing high precision in the acquire data. Quality indicators habitually established were used: %Col (>1), %C (30-44%), %N (11-16%), %S (0.15-0.35%), C:N (2,9-3,6), C:S (600 ± 300) and N:S (200 ± 100)[18–21].
Dental calculus analysis. Calculus samples were taken from 25 teeth belonging to 16 of the total of 20 individuals from El Espinoso cave. The dental calculus matrix was removed from the teeth following the protocol by Sabin and Fellow[22] using a disposable blade and wearing powder-free gloves to prevent contamination. Calculus samples were stored in sterile Eppendorf tubes. Decontamination and the extraction procedures for micro-debris were carried out according to standard protocols as described by Cristiani et al.[23,24], and they were conducted in dedicated clean spaces under strict environmental monitoring of the DANTE – Diet and Ancient Technology – laboratory of Sapienza University of Rome. Dental calculus decontamination was carried out under the microscope with magnification up to 100x. Soil particles adhered to the plaque's surface was scratched using a sterile acupuncture needle with 0.06 N HCl for cleaning. Once clean, calculus samples were washed in ultrapure water up to three times. Later, calculus was degraded in a weak solution of 0.06 N HCl with the aim to extract the microfossils entrapped in the calculus matrix. The dissolved calculus was mounted on slides using a solution of 50:50 glycerol and ultrapure water.

Samples of the sediment removed from dental calculus and the burial area of the cave were also analyzed and compared with the results obtained on the human calculus. Bench working areas and dust traps placed in the laboratory were also regularly checked as control samples. The analysis of the microfossils was carried out using a Zeiss Imager2 polarized microscope (100x–630x) at the DANTE laboratory and a Leica DVM6 M digital microscope at the EvoAdapta Group. A reference collection of more than 300 modern plants from southwestern Europe housed at DANTE laboratory was used as comparison. In addition, a reference collection of more than 100 modern species from northern Spain was created for a more local approach using the archaeobotanical and palynological data about the flora and agriculture of the Cantabrian Region during the Holocene. They were collected by Grupo EvoAdapta Group and housed at the University of Cantabria[25–30]. Further statistical work on modern seeds was carried out in order to identify at the species level the archaeological starch granules previously assigned to the *Trititiceae* and *Paniceae* tribes. Caryopses from six species belonging to the *Triticeae* tribe (*Hordeum vulgare, Secale cereale, Triticum aestivum, Triticum dicoccoides, Triticum dicoccum and Triticum monococcum*) and five species belonging to the *Paniceae* tribe (*Panicum miliaceum, Setaria italica, Setaria verticillata, Setaria viridis and Shorghum halepensis*) were collected and compared among them. Seeds were grounded using pestle and mortar. Starch powder (0.5 mg) was resuspended in 100 µL of sterile distilled water and vortexed for 5 minutes. Later, the sample was observed by an optic transmitted light microscopy. One hundred starch granules were selected, and their length measured. Minimum and maximum lengths, mean, and median values with relative standard deviations and their IQR ranges were reported for each species in Supplementary Table 4 and plotted in Supplementary Fig. 9.

Supplementary Figures 1 to 9

Supplementary Figure 1. A) El Espinoso cave plan. B) Limestone cliff whereby access to the site (Scale bar: 1 m). C) Location of the human bones scattered over the cave floor surface (Scale arrow: 10 cm). The base plan (A) was elaborated using Adobe Illustrator software (v16.0.3. https://www.adobe.com/es/products/illustrator/) and further editing with Apple Preview software (v10.0 https://support.apple.com/es-es/guide/preview/welcome/mac).

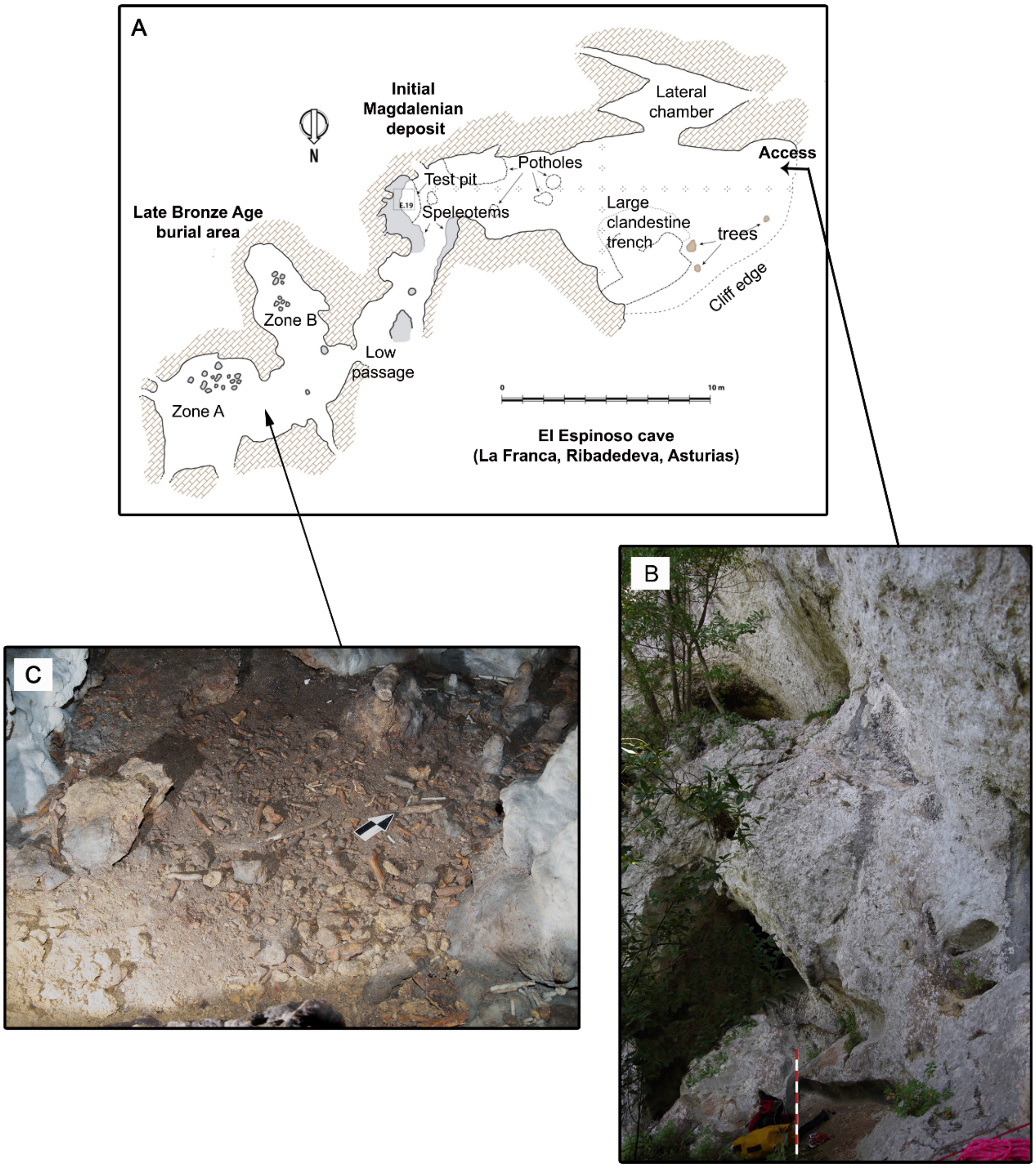

Supplementary Figure 2. A) La Llana human skeleton cemented to the floor cave (Scale bars: 10 cm). B) Human bones recovered during the excavation.

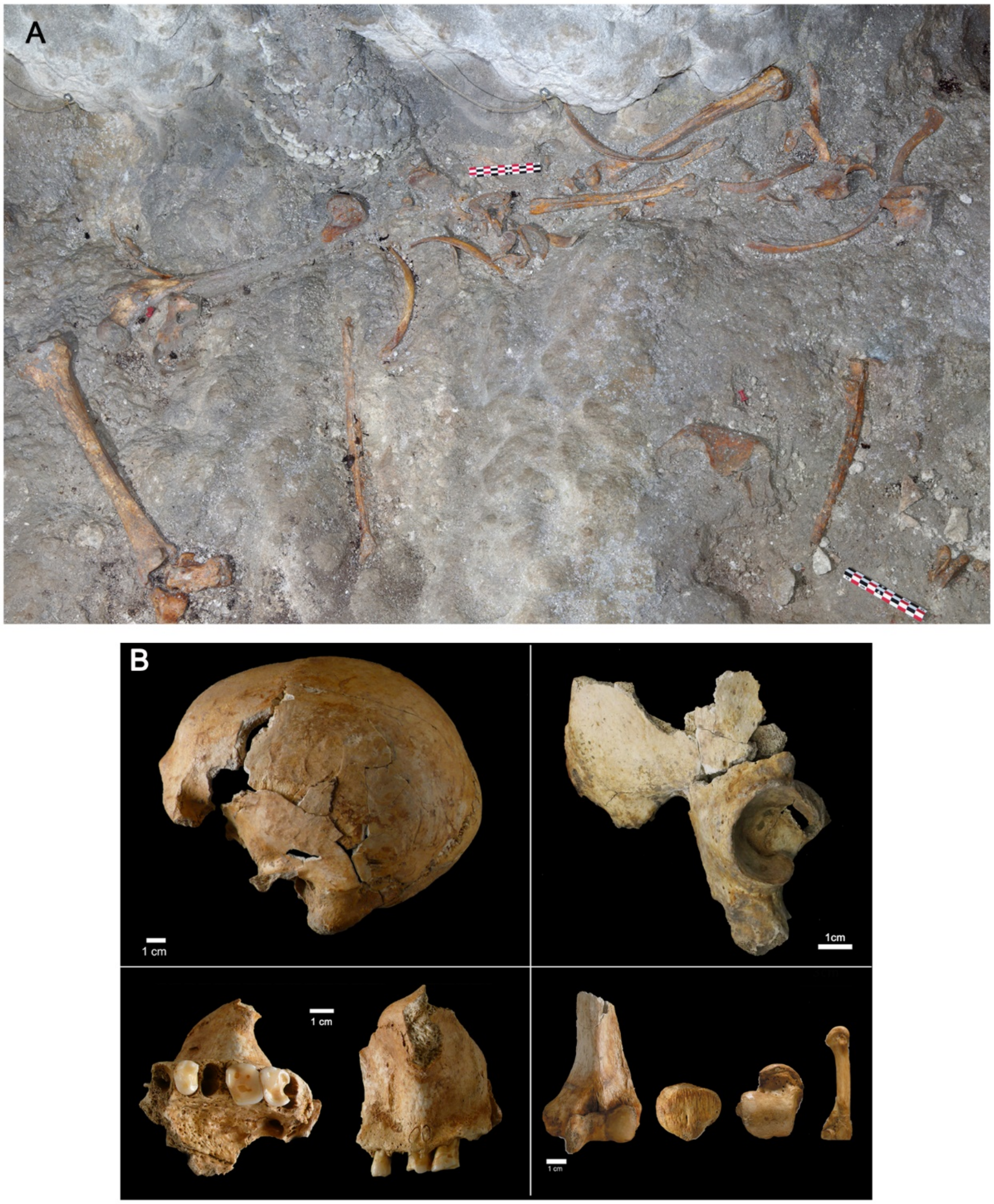

Supplementary Figure 3. A) View of La Paré de Los Cinchos mountain and B) Plan and elevation model of Los Cinchos cave by Interclub Ubiña-Colectivo Asturiano de Espeleólogos. C) Location of the skeleton at the bottom of the karstic system by César García de Castro y Valdés and Gabino Busto Hevia.

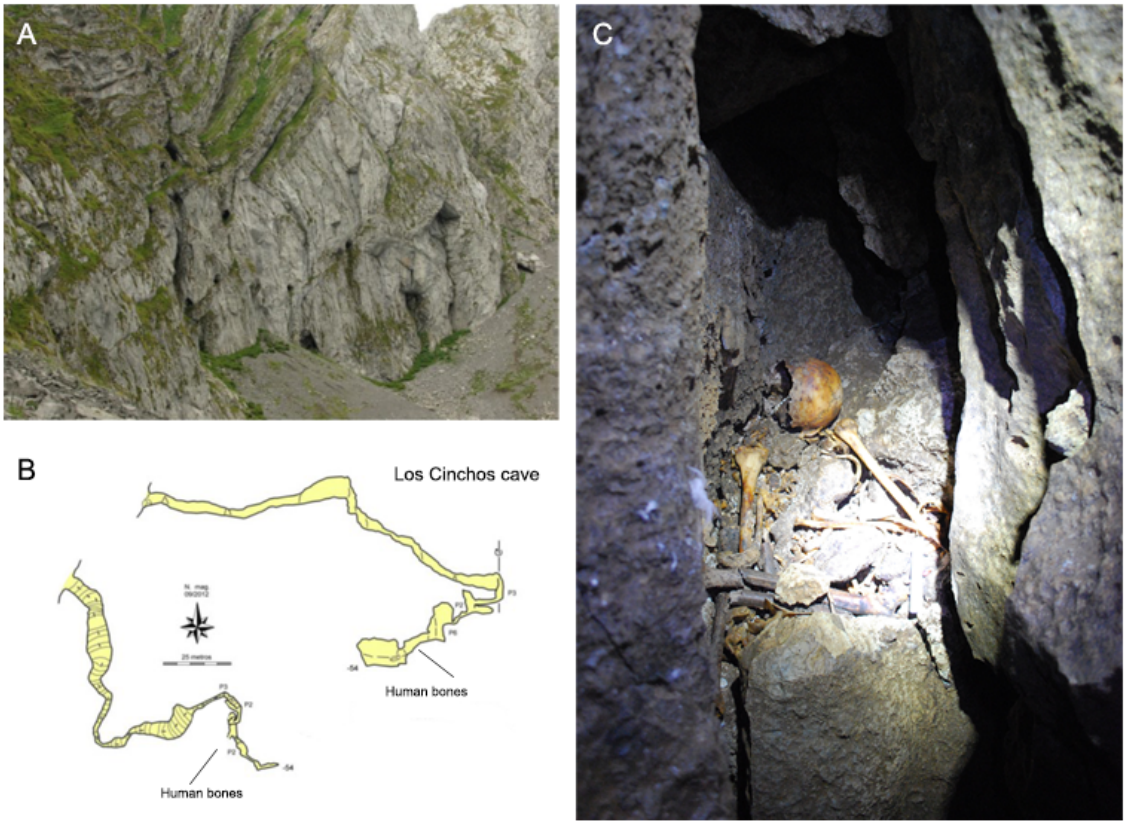

Supplementary Figure 4. A) Stratigraphic sequence of La Fragua cave (Scale bar: 1 m). B) Human bones recovered from Trench. C) Slab at the bottom of the trench (Scale bar: 40 cm).

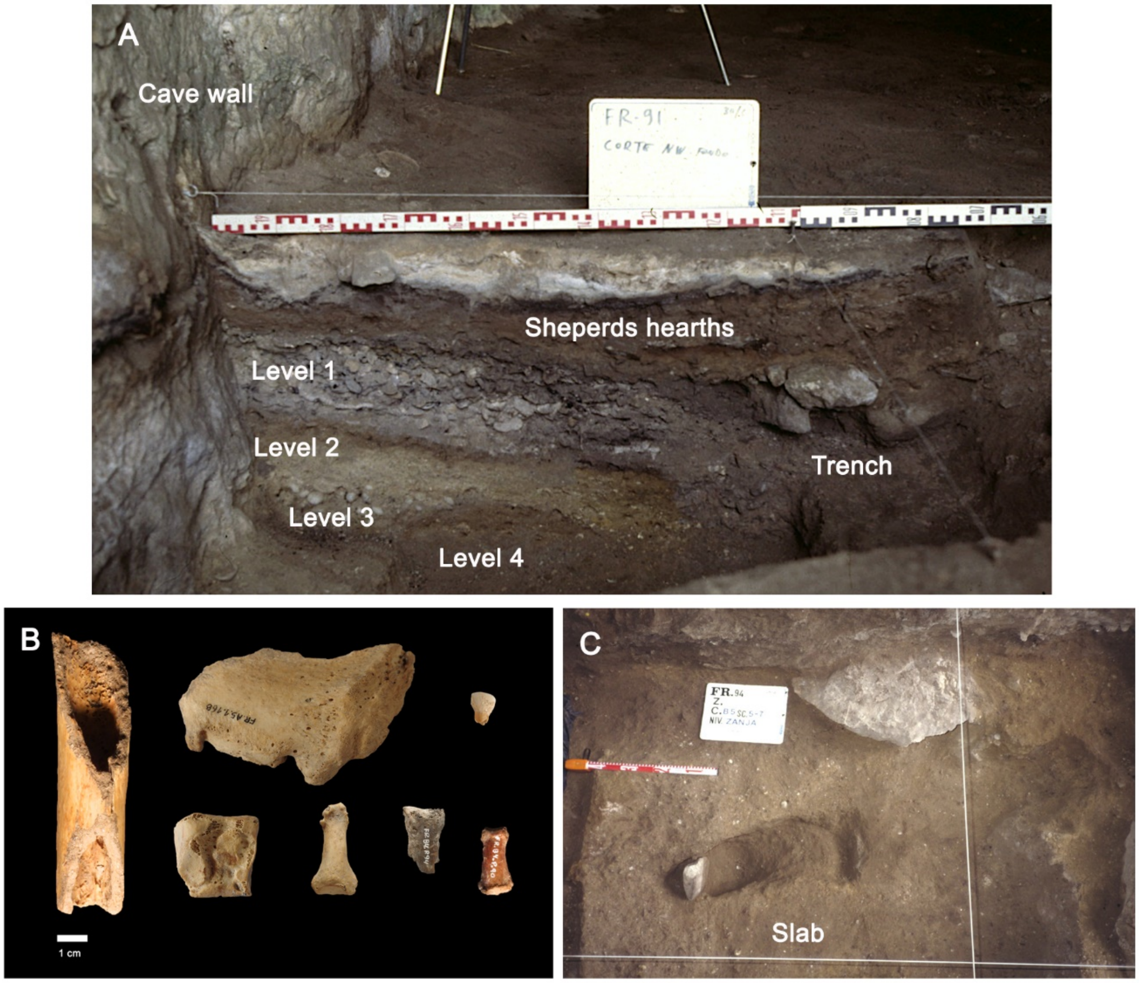

Supplementary Figure 5. Experimental starch grains of *Panicum miliaceum*. Starch granules seen in light microscope and polarized light microscope. (Scale bar, 20 µm).

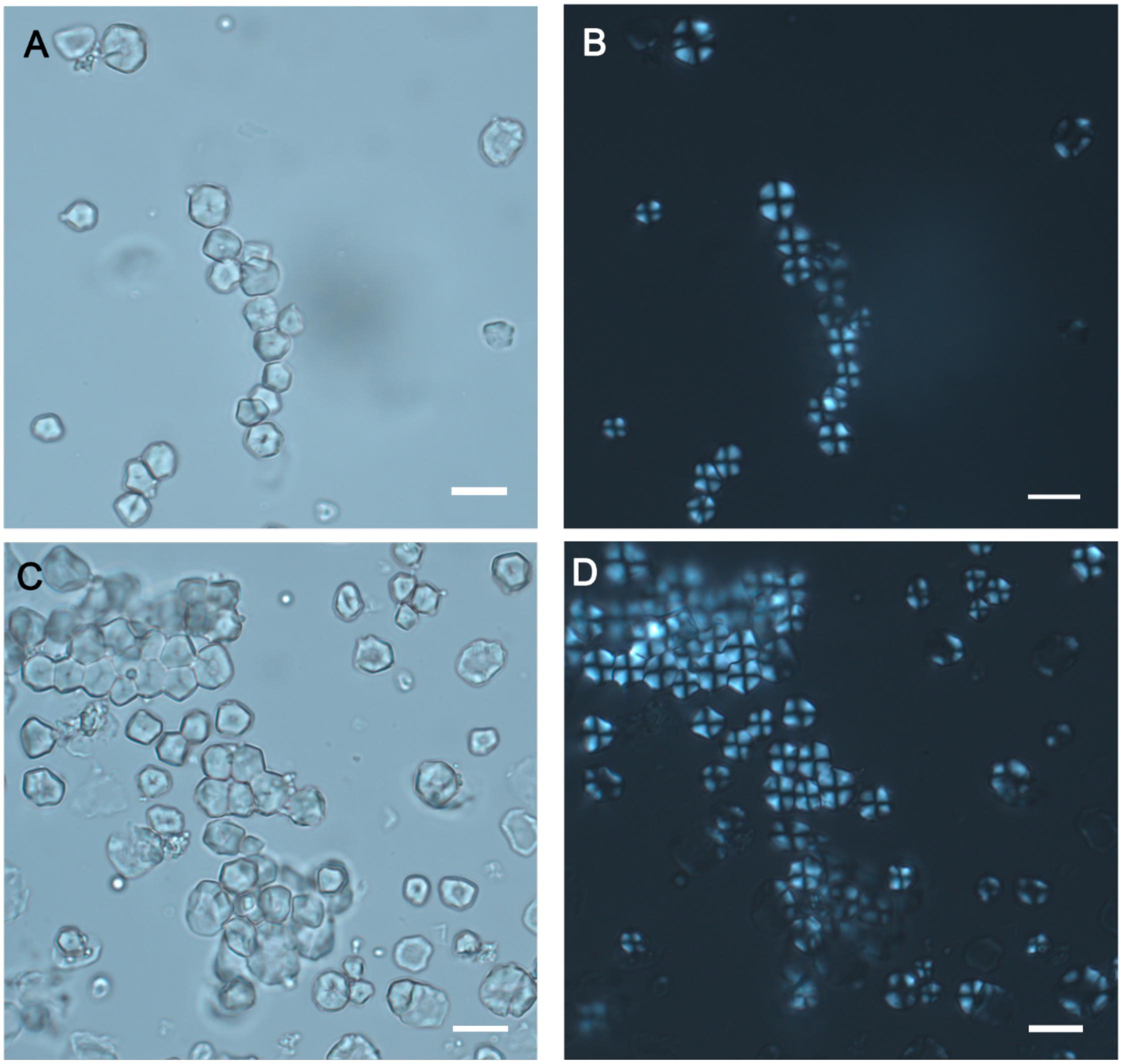

Supplementary Figure 6. Experimental starch grains of *Setaria italica.* Starch granules seen in light microscope and polarized light microscope. (Scale bar, 20 μm).

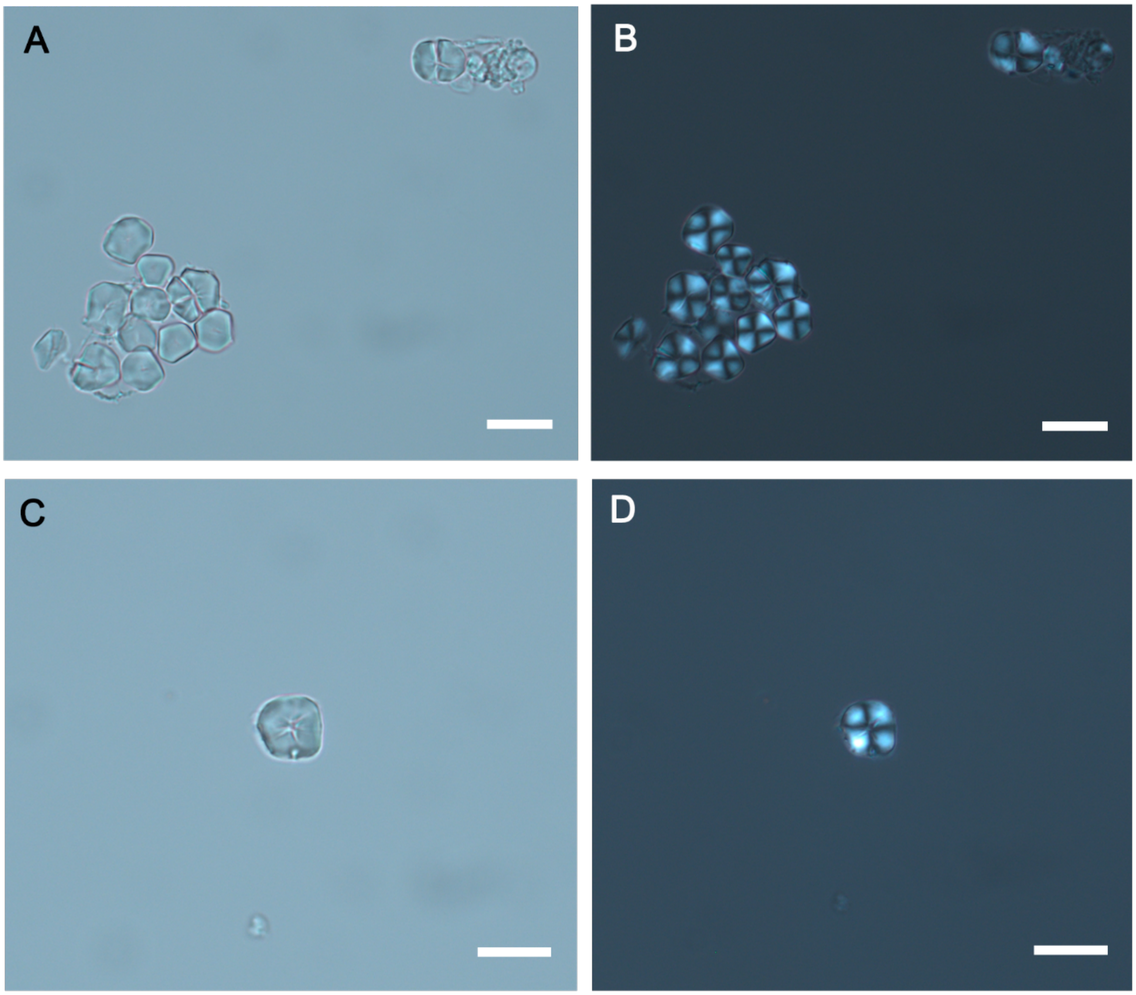

Supplementary Figure 7. Experimental starch grains from different species of Triticeae tribe. (A-B) *Triticum aestivum*. (C-D) *Triticum monococcum*. (E-F) *Triticum dicoccum*. (G-H) *Triticum dicoccoides*. (I-J) *Hordeum vulgare*. (K-L) *Secale cereale*. Starch granules in light microscope and polarized light microscope. (Scale bar, 20 μm).

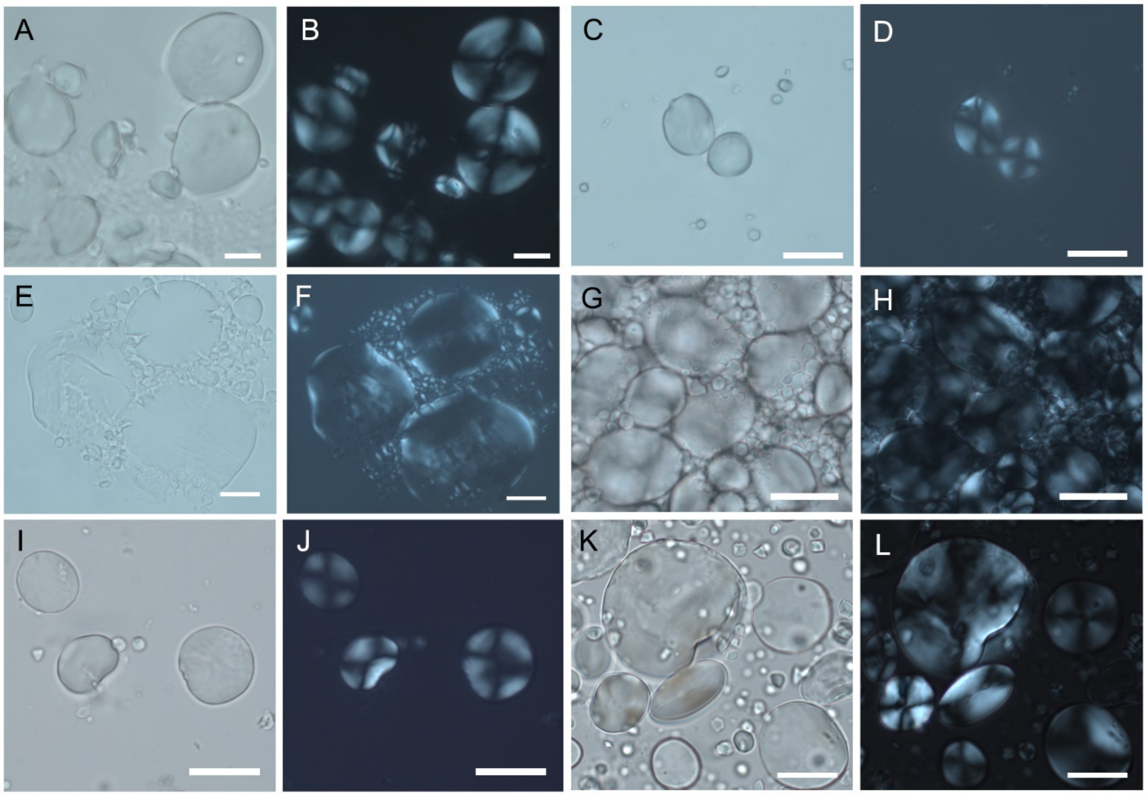

Supplementary Figure 8. Non-dietary micro-debris identified in El Espinoso dental plaque: A-B) Fungal spores. C) Wood remains next to dental calculus. D) The same wood remains in polarized light. E) Vessel member. F) Conifer pollen grain. G) Fiber and charcoals remains entrapped within the calculus matrix. H) Vegetal tissue. (Scale bar, 20 µm).

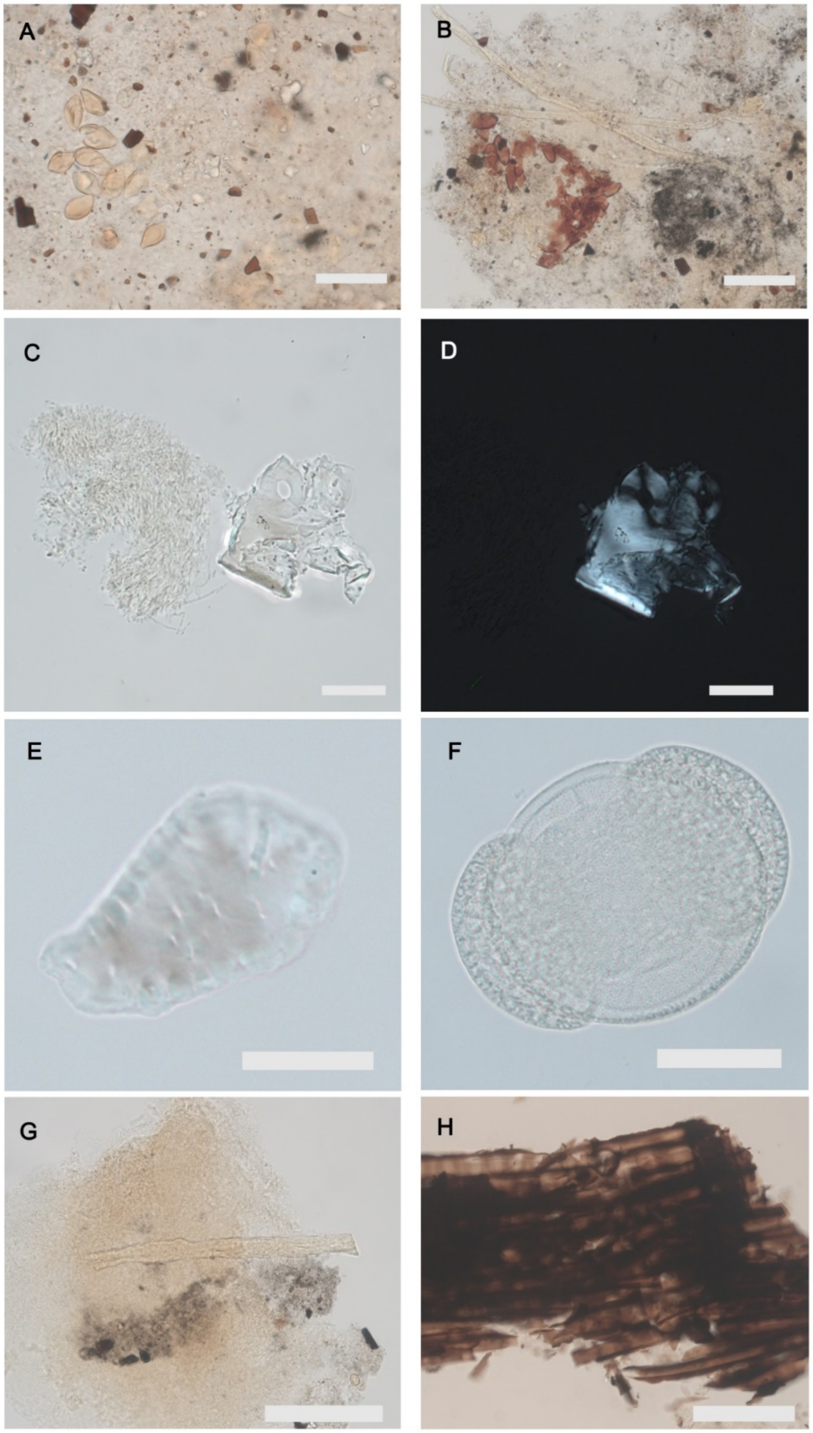

Supplementary Figure 9. Violin plot of comparing the length of starch granules in modern cereal species (Length given in μm).

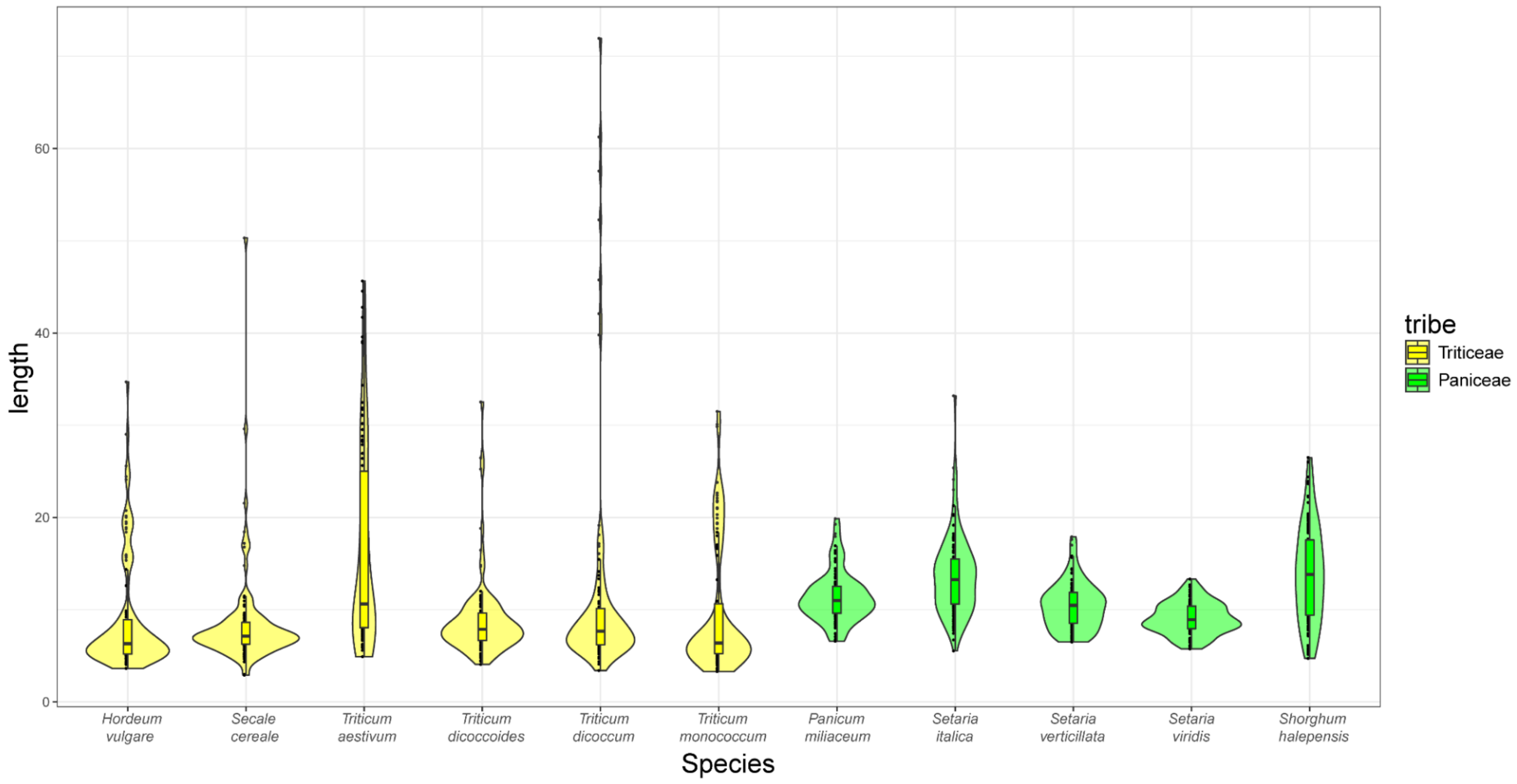

Supplementary Tables 1 to 4

Supplementary Table 1. AMS dates from Bronze Age sites discussed in the text. Samples were directly performed on human bones. Radiocarbon dates were calibrated in OxCal v4.4 using the IntCal20 calibration curve[14]. All results are presented at a 95.4% probability. EBA: Early Bronze Age; MBA: Middle Bronze Age; LBA: Late Bronze Age.

Site	Lab code	SUC number	Sample	Lab reference	Date BP	Date cal. BC	Cultural period	References
El Espinoso	ES00	-	Tooth	ICA-14T/0804	2960 ± 40	1369-1019	LBA	5
	ES01	SUC 341	Ulna	OxA-38397	2935 ± 20	1216-1052	LBA	This paper
	ES02	SUC 342	Ulna	OxA-38398	2962 ± 20	1262-1112	LBA	This paper
	ES03	SUC 343	Ulna	OxA-38660	2898 ± 22	1198-1008	LBA	This paper
	ES04	SUC 344	Ulna	OxA-38399	2920 ± 20	1210-1019	LBA	This paper
	ES05	SUC 345	Ulna	OxA-38400	2977 ± 20	1267-1122	LBA	This paper
	ES06	SUC 346	Ulna	OxA-38401	2907 ± 21	1201-1013	LBA	This paper
	ES07	SUC 347	Ulna	OxA-38402	2936 ± 20	1216-1052	LBA	This paper
	ES08	SUC 348	Ulna	OxA-38403	2979 ± 20	1269-1122	LBA	This paper
	ES09	SUC 349	Ulna	OxA-38404	2964 ± 20	1262-1115	LBA	This paper
	ES10	SUC 350	Ulna	OxA-38405	2922 ± 20	1211-1021	LBA	This paper
	ES11	SUC 351	Ulna	OxA-38406	2930 ± 20	1214-1050	LBA	This paper
	ES12	SUC 352	Ulna	OxA-38407	2917 ± 20	1207-1018	LBA	This paper
	ES13	SUC 353	Ulna	OxA-38408	2961 ± 35	1285-1048	LBA	This paper
	ES14	SUC 354	Ulna	OxA-38409	2970 ± 20	1263-1121	LBA	This paper
La Llana	LL01	SUC 357	Coxal	UGAMS-9083	3300 ± 25	1618-1510	MBA	8
Los Cinchos	CI01	SUC 367	Human bone	Beta-366545	3550 ± 30	2014-1771	EBA	9
La Fragua	FR01	S-UC 443	Humerus	OxA-31057	3697 ± 30	2199-1978	EBA	13

Supplementary Table 2. Human and animal isotopic values from El Espinoso, La Llana, Los Cinchos and La Fragua caves.

Lab code	SUC number	Specie	Sample	Age	$\delta^{13}C_{V\text{-}PDB}$	$\delta^{15}N_{AIR}$	$\delta^{34}S_{V\text{-}CDT}$	Yield (%)	%C	%N	%S	C:N	C:S	N:S
ES01	SUC 341	*Homo sapiens*	Ulna	Adult	-15	10.4	15.8	7.9	41.6	15.4	0.19	3.2	583	184
ES02	SUC 342	*Homo sapiens*	Ulna	Adult	-16.5	9.3	14.3	13.5	42.3	15.7	0.20	3.1	567	181
ES03	SUC 343	*Homo sapiens*	Ulna	Adult	-15.2	9.3	14.8	13.8	45	16.6	0.21	3.2	563	178
ES04	SUC 344	*Homo sapiens*	Ulna	Subadult	-14	9.5	16.9	13.1	45.5	17	0.21	3.1	580	186
ES05	SUC 345	*Homo sapiens*	Ulna	Adult	-15.5	9.3	14.4	14.4	43.7	16.1	0.21	3.2	557	176
ES06	SUC 346	*Homo sapiens*	Ulna	Adult	-15	10	14.8	7.6	37.9	13.8	0.23	3.2	442	138
ES07	SUC 347	*Homo sapiens*	Ulna	Adult	-15.7	9.7	15.1	11.2	45.5	16.8	0.20	3.2	603	191
ES08	SUC 348	*Homo sapiens*	Ulna	Adult	-16.6	9.4	14	8.6	43.5	16.1	0.22	3.1	535	170
ES09	SUC 349	*Homo sapiens*	Ulna	Adult	-14.9	9.7	14.6	14.5	44.7	16.5	0.21	3.2	557	177
ES10	SUC 350	*Homo sapiens*	Ulna	Subadult	-15	9.2	14.3	8.6	38.2	14	0.20	3.2	522	164
ES11	SUC 351	*Homo sapiens*	Ulna	Subadult	-14.7	9.1	14.6	11	44.1	16.2	0.22	3.2	525	165
ES12	SUC 352	*Homo sapiens*	Ulna	Adult	-17.8	9.2	13.9	8.4	43.3	15.9	0.22	3.2	516	163
ES13	SUC 353	*Homo sapiens*	Ulna	Subadult	-17.1	10.1	15.3	10.9	47.5	17.6	0.23	3.2	547	173
ES14	SUC 354	*Homo sapiens*	Ulna	Adult	-15.4	9.5	15.5	6.4	38.7	14.2	0.20	3.2	522	164
ES15	SUC 355	*Bos taurus*	Metacarpus	Adult	-22.1	4.3	16.2	9.9	44.8	16.7	0.18	3.1	653	208
ES16	SUC 356	*Ovis aries*	Rib	Adult	-20.6	3.7	13.5	5.3	40.1	14.5	0.15	3.2	730	227
LL01	SUC 357	*Homo sapiens*	Coxal	Adult	-21.1	9.2	14.5	8.2	44.4	16.1	0.22	3.2	535	166
CI01	SUC 367	*Homo sapiens*	Rib	Adult	-20.9	8.6	14.9	15.3	39.3	15	0.20	3.1	515	168
FR01	S-UC 443	*Homo sapiens*	Humerus	Adult	-20.4	9.2	14.8	14.7	42.9	15.5	0.21	3.2	539	167

Supplementary Table 3. Summary data of the individuals analyzed for dental calculus analysis from El Espinoso cave.

Individual	Age	Tooth	Quantity	2D/3D morphology	Hillum	Lamellae	Identification	Sample weight (mg)
N1 60	Adult	ULP^4	2	3D polyhedral shape	Central, distinctive cross extinction	Not visible	Paniceae	0.2
N3 37	Adult	ULM^1	8	3D polyhedral shape	Central, with fissures radiating from it, distinctive cross extinction	Not visible	Paniceae	0.4
			1	Round to oval in 2D view	Central, slightly sunken	Not visible	Triticeae	
N3 40, 41, 43	Infant	ULdi^1, URdi^1, LRdi^2	1	Round to oval in 2D view	Central, slightly sunken	Not visible	Triticeae	7.7
N5 3	Adult	ULI^1	1	3D polyhedral shape	Central, with fissures radiating from it, distinctive cross extinction	Not visible	Paniceae	0.9
N6 3	Adult	LRP^3	1	Oval in 2D view	Central, slightly sunken	Not visible	Triticeae	0.2
			3	Round to oval in 2D view	Central, slightly sunken	Present, concentrated around the hilum	Triticeae	
N7 1	Adult	LRM^2	3	Round to oval in 2D view	Central, slightly sunken	Present, concentrated around the hilum	Triticeae	6.3
			12	Round to oval in 2D view	Central, distinctive cross extinction	Not visible	Triticeae	
R18 75, 77, 78, 79	Adult	LRI^1, LRI^2, LRP^4, LLP^4	0					3.5
S20 14, 17, 19	Adult	LLP^3, LLM^1, LRI^2	3	3D polyhedral shape	Central, with fissures radiating from it, distinctive cross extinction	Not visible	Paniceae	0.6
			3	Round to oval in 2D view	Central	Present, concentrated around the hilum	Triticeae	
T20 70	Adult	LRI^1	1	Round to oval in 2D view	Central, slightly sunken	Not visible	Triticeae	0.6
T20 67, 74	Adult	LLM^1, LLM^2	2	3D polyhedral shape	Central, distinctive cross extinction	Not visible	Paniceae	0.7
T20 78	Adult	LRI^1	4	Round to oval in 2D view	Central, slightly sunken	Barely visible	Triticeae	2.8
U20 15	Adult	LRC	5	Round to oval in 2D view	Central	Present, concentrated around the hilum	Triticeae	0.7
V18 67	Adult	LRM^2	4	3D polyhedral shape	Central, with fissures radiating from it, distinctive cross extinction	Not visible	Paniceae	1.6
			1	Round to oval in 2D view	Central, slightly sunken	Not visible	Triticeae	
V19 20	Adult	LRM^1	5	Round to oval in 2D view	Central	Present, concentrated around the hilum	Triticeae	0.9
V21 6	Adult	LLM^1	9	3D polyhedral shape	Central, with fissures radiating from it, distinctive cross extinction	Not visible	Paniceae	3.1
			2	Round to oval in 2D view	Central	Not visible	Triticeae	
W18 24	Adult	URP^4	3	Round to oval in 2D view	Central, slightly sunken	Not visible	Triticeae	3.5

Supplementary Table 4. Summary statistics of the length of starch granules from modern seeds belonging to the *Triticeae* and *Paniceae* tribes.

Species	Min.	Max.	Mean	Median	StDev.	IQR
Triticeae						
Hordeum vulgare	3,63	34,71	8,95	6,30	6,23	3,74
Secale cereale	2,89	50,31	8,45	7,14	5,63	2,38
Triticum aestivum	4,90	45,64	16,66	10,62	10,95	16,95
Triticum dicoccoides	4,07	32,52	8,78	7,87	4,23	2,96
Triticum dicoccum	3,40	71,95	11,37	7,66	12,22	3,93
Triticum monococcum	3,28	31,50	9,58	6,38	6,98	5,40
Paniceae						
Panicum miliaceum	6,56	19,87	11,36	10,98	2,74	2,94
Setaria italica	5,53	33,19	13,61	13,27	4,28	4,89
Setaria verticillata	6,48	17,90	10,39	10,48	2,52	3,32
Setaria viridis	5,74	13,31	9,07	8,91	1,72	2,46
Sorghum halepensis	4,72	26,50	13,91	13,83	5,27	8,12

Supplementary References

1. González Morales, M. R. Memoria de los trabajos de limpieza y toma de muestras en los yacimientos de las cuevas de Mazaculos y El Espinoso (La Franca, Ribadedeva) y La Llana (Andrín, Llanes) en 1993. in *Excavaciones Arqueológicas en Asturias, 1991-94* 65–78 (Gobierno del Principado de Asturias, 1995).
2. Cuenca-Solana, D. Utilización de instrumentos de concha para la realización de actividades productivas en las formaciones económico sociales de los cazadores-recolectores-pescadores y primeras sociedades tribales de la fachada atlántica europea. (Tesis Doctoral Inédita, Universidad de Cantabria, 2012).
3. González-Rabanal, B., González Morales, M. R. & Marín-Arroyo, A. B. Anthropological and taphonomical study of human remains from the burial cave of El Espinoso (Ribadedeva, Asturias, Spain). in *Current Approaches to Collective Burials in the Late European Prehistory* (eds. Tomé, T., Díaz-Zorita Bonilla, M., Silva, A. M., Cunha, C. & Boaventura, R.) 55–65 (Archaeopress, 2017).
4. González-Rabanal, B., González-Morales, M. R. & Marín-Arroyo, A. B. La tafonomía como marco metodológico para interpretar depósitos funerarios superficiales: estudio de la cueva sepulcral de El Espinoso (Ribadedeva, Asturias). *Trab. Prehist.* **74**, 278–295 (2017).
5. González-Rabanal, B. Estudio arqueológico y tafonómico de los restos humanos de la cueva sepulcral de El Espinoso (Ribadedeva, Asturias). (Trabajo Fin de Máster Inédito, Universidad de Cantabria, 2014).
6. Pérez Suárez, C. Carta Arqueológica de los concejos de Llanes y Ribadedeva (1992). in *Excavaciones Arqueológicas en Asturias, 1991-94* 243–245 (Gobierno del Principado de Asturias, 1995).
7. Estalrrich, A., González-Rabanal, B., Marín-Arroyo, A. B., Maeso, C. V. & González Morales, M. R. Osteolytic lesions on the os petrosum of a Bronze Age individual from La Llana cave (Northern Spain) compatible with a possible case of otitis media. A multifaceted methodological approach. *International Journal of Paleopathology* **31**, 97–102 (2020).

8. Vega Maeso, C. La cerámica inciso-impresa en el tránsito del III al II milenio cal B.C. en la Región Cantábrica. (Tesis Doctoral Inédita, Universidad de Cantabria, 2015).
9. García de Castro, C. & Busto Hevia, G. Hallazgo y extracción de un esqueleto humano de la Edad del Bronce en la cueva de la Paré los Cinchos (Puertu Güeria, Quirós, Asturias). in *Excavaciones Arqueológicas en Asturias, 2013-2016* 183–192 (Gobierno del Principado de Asturias, 2018).
10. Alonso-Llamazares, C. & López Martínez, B. Estudio antropológico de los restos óseos humanos recuperados en el macizo de Ubiña. in *Excavaciones Arqueológicas en Asturias, 2013-2016* 193–204 (Gobierno del Principado de Asturias, 2018).
11. González Morales, M. R. & Díaz Casado, Y. La Prehistoria de las Marismas: excavaciones arqueológicas en los abrigos de la Peña del Perro, Santoña. in *Actuaciones Arqueológicas en Cantabria, 1984-1999* (Gobierno de Cantabria, 2000).
12. Marín-Arroyo, A. B. Análisis arqueozoológico, tafonómico y de distribución espacial de la fauna de mamíferos de la Cueva de la Fragua (Santoña, Cantabria). *Munibe Antropología-Arkeología* **56**, 19–44 (2004).
13. González Morales, M. R. La Prehistoria del Valle del Asón: un resumen de 30 años de investigaciones. *Sautuola* **XX**, 53–72 (2015).
14. Reimer, P. J. *et al.* The IntCal20 Northern Hemisphere Radiocarbon Age Calibration Curve (0–55 cal kBP). *Radiocarbon* **62**, 725–757 (2020).
15. Bronk Ramsey, C. Bayesian Analysis of Radiocarbon Dates. *Radiocarbon* **51**, 337–360 (2009).
16. Bronk Ramsey, C. Methods for Summarizing Radiocarbon Datasets. *Radiocarbon* **59**, 1809–1833 (2017).
17. Richards, M. P. & Hedges, R. E. M. Stable Isotope Evidence for Similarities in the Types of Marine Foods Used by Late Mesolithic Humans at Sites Along the Atlantic Coast of Europe. *Journal of Archaeological Science* **26**, 717–722 (1999).
18. van Klinken, G. J. Bone Collagen Quality Indicators for Palaeodietary and Radiocarbon Measurements. *J. Archaeol. Sci.* **26**, 687–695 (1999).

19. DeNiro, M. J. Postmortem preservation and alteration of in vivo bone collagen isotope ratios in relation to palaeodietary reconstruction. *Nature* **317**, 806–809 (1985).

20. Ambrose, S. H. Preparation and characterization of bone and tooth collagen for isotopic analysis. *Journal of Archaeological Science* **17**, 431–451 (1990).

21. Nehlich, O. & Richards, M. P. Establishing collagen quality criteria for sulphur isotope analysis of archaeological bone collagen. *Archaeol. Anthropol. Sci.* **1**, 59–75 (2009).

22. Sabin, S. & James, A. Dental Calculus Field-Sampling Protocol (Sabin version) v2. *(protocols.io.bqecmtaw)* http://dx.doi.org/10.17504/protocols.io.bqecmtaw (2020) doi:10.17504/protocols.io.bqecmtaw.

23. Cristiani, E., Radini, A., Edinborough, M. & Borić, D. Dental calculus reveals Mesolithic foragers in the Balkans consumed domesticated plant foods. *Proc. Natl. Acad. Sci. U. S. A.* **113**, 10298–10303 (2016).

24. Cristiani, E. *et al.* Dental calculus and isotopes provide direct evidence of fish and plant consumption in Mesolithic Mediterranean. *Sci. Rep.* **8**, 8147 (2018).

25. Zapata, L. La explotación de los recursos vegetales y el origen de la agricultura en el País Vasco. (Tesis Doctora Inédita, Universidad del País Vasco, 2002).

26. Peña-Chocarro, L., Zapata, L., Iriarte, M. J., González Morales, M. & Straus, L. G. The oldest agriculture in northern Atlantic Spain: new evidence from El Mirón Cave (Ramales de la Victoria, Cantabria). *J. Archaeol. Sci.* **32**, 579–587 (2005).

27. López-Merino, L., Cortizas, A. M. & López-Sáez, J. A. Early agriculture and palaeoenvironmental history in the North of the Iberian Peninsula: a multi-proxy analysis of the Monte Areo mire (Asturias, Spain). *J. Archaeol. Sci.* **37**, 1978–1988 (2010).

28. López-Dóriga, I. The use of plants during the Mesolithic and the Neolithic in the Atlantic coast of the Iberian peninsula. (Tesis Doctoral Inédita, Universidad de Cantabria, 2016).

29. Núñez de la Fuente, S. Dinámicas socio-ecológicas, resiliencia y vulnerabilidad en un paisaje atlántico montañoso: la Región Cantábrica durante el Holoceno. (Tesis Doctoral Inédita, Universidad de Cantabria, 2018).

30. Tereso, J. P. *et al.* Agriculture in NW Iberia during the Bronze Age: A review of archaeobotanical data. *Journal of Archaeological Science: Reports* **10**, 44–58 (2016).
